# The Impact of Genetics on Pediatric Interstitial Lung Diseases: A Narrative Literature Review and Clinical Implications

**DOI:** 10.3390/biomedicines14020385

**Published:** 2026-02-06

**Authors:** Martina Mazzoni, Sonia Lomuscio, Adriano La Vecchia, Rosamaria Terracciano, Fabio Antonelli, Pierluigi Vuilleumier, Annalisa Allegorico

**Affiliations:** 1 Department of Medicine, Surgery and Dentistry “Scuola Medica Salernitana”, Pediatrics Section, University of Salerno, 84081 Baronissi, Italy; martmazz94@gmail.com; 2 Department of Biomedicine and Prevention, University of Rome Tor Vergata, 00133 Rome, Italy; sonia.lomuscio@students.uniroma2.eu; 3 Pediatric Unit, Fondazione IRCCS (Istituto di Ricovero e Cura a Carattere Scientifico) Ca’ Granda Ospedale Maggiore Policlinico, 20122 Milan, Italy; 4 Department of Medicine and Surgery, University of Milan-Bicocca, 20122 Milan, Italy; 5 Department of Translational Medicine, Section of Pediatrics, University of Naples “Federico II”, 80129 Naples, Italy; rosamariaterracciano@libero.it; 6 Unit of Pediatric Pneumology and UTSIR (Unità di Terapia Sub-Intensiva Respiratoria), Santobono-Pausilipon Children’s Hospital, 80129 Naples, Italy; fabantonelli65@gmail.com (F.A.); p.vuilleumier@santobonopausilipon.it (P.V.)

**Keywords:** chILD, NGS, pulmonary fibrosis, genotype–phenotype correlation, surfactant dysfunction, telomere biology disorders, precision medicine

## Abstract

**Background**: Interstitial lung diseases (ILDs) are a heterogeneous group of disorders characterized by variable degrees of inflammation and fibrosis affecting the pulmonary interstitium. Advances in molecular biology and genetics have greatly expanded our understanding of ILD pathogenesis, uncovering novel mechanisms and supporting precision medicine approaches. **Genetic Insights**: Genetic factors play a pivotal role in ILD heterogeneity, influencing disease onset, severity, and progression. To date, more than 30 genes with different inheritance patterns (autosomal dominant, recessive, or X-linked) have been associated with ILDs. These genes are primarily involved in surfactant metabolism, telomere maintenance, immune regulation, and epithelial repair. Emerging evidence also implicates genes encoding aminoacyl-tRNA synthetases. This review summarizes the main genetic alterations underlying ILD pathogenesis and discusses their impact on diagnostic and therapeutic approaches, highlighting how identification of disease-causing variants can improve diagnostic accuracy, refine prognostic assessment, and inform recurrence risk. **Methods**: A narrative review was conducted through targeted PubMed and Embase searches using disease- and gene-related keywords. Studies were prioritized based on predefined conceptual criteria, including clinical relevance, strength and replication of genetic associations, and availability of functional or translational evidence. **Conclusions**: This synthesis brings together the latest genetic insights into pediatric ILDs and their clinical implications. Integrating genomic data into clinical practice may enable earlier diagnosis, tailored follow-up, individualized therapeutic strategies, and more informed genetic counseling. However, important challenges remain, including incomplete genotype–phenotype correlations and limited functional validation for several disease-associated genes, which currently constrain full clinical translation.

## 1. Introduction

### 1.1. Brief Overview of Pediatric Interstitial Lung Diseases (ILDs)

Interstitial lung diseases (ILDs) represent a broad spectrum of pulmonary disorders characterized by varying degrees of inflammation and fibrosis affecting the lung interstitium, ultimately leading to progressive dyspnea and, in many cases, end-stage respiratory failure [1]. Although ILDs are well studied in adults, pediatric forms represent a distinct subgroup, differing in etiology, pathophysiology, and clinical presentation due to the developmental dynamics of the growing lung. In pediatrics, the acronym chILD (children’s ILD) is widely used to distinguish these disorders from ILD in adults.

Classification frameworks for ILDs are continuously updated as new clinical, imaging, histopathological, and genetic evidence emerges; therefore, they should be regarded as dynamic tools, whose applicability to pediatric populations evolves in parallel with advances in molecular diagnostics and disease characterization. With more than 200 conditions encompassed within the chILD spectrum, classification remains a matter of debate, ranging from broader grouping strategies to the recognition of discrete disease entities. Proponents of a unified approach conceptualize chILD as disorder of abnormal lung development that may be unmasked by a “second hit” (e.g., infection), whereas advocates of a more granular model emphasize distinct, genetically defined disease entities [2].

In adults, early classifications were primarily histopathological and identified four subgroups: usual interstitial pneumonia, desquamative interstitial pneumonia (DIP) and its related form, respiratory bronchiolitis–associated ILD, acute interstitial pneumonia (formerly Hamman–Rich syndrome), and non-specific interstitial pneumonia (NSIP). Over time, a multidisciplinary approach replaced this histopathologic model, and specific entities were validated through the international ATS/ERS consensus statements on idiopathic interstitial pneumonias. Similarly, chILD classifications initially derived from adult histopathological schemes, have progressively shifted toward multidisciplinary models that integrate age, clinical context, biological data, high-resolution computed tomography (HRCT) findings, and lung tissue analysis [3].

Traditionally, classification frameworks have separated disorders according to age. The framework proposed by Nathan et al. in 2020 [2] emphasizes developmental stage, distinguishing ILDs specific to infancy (<2 years) (e.g., NEHI, pulmonary interstitial glycogenosis, developmental disorders) from non–age-specific pediatric ILDs, including immune-mediated, environmental, and parenchymal dysfunctions. However, Cunningham et al. [2] note that while age-based classification is conceptually useful, it is increasingly limited in practice, particularly for genetically diagnosed conditions spanning overlapping age ranges. In this context, The Nathan et al. model uses developmental stage as a clinical guide, whereas the Cunningham et al. framework shifts toward genotype-driven and pathophysiology-based classification, integrating genetic, radiologic, and clinical features beyond age.

Griese (2022) [3], in *Etiologic Classification of Diffuse Parenchymal (Interstitial) Lung Diseases*, summarizes the evolution of ILD classification systems in both pediatric and adult pneumology and outlines the etiologic framework adopted in the chILD-EU registry, which groups ILDs into lung-only, systemic disease–related, exposure-related, and vascular disorders. Similarly, Maher et al. propose an etiologic categorization of interstitial lung diseases that includes connective tissue disease–associated ILD (CTD-ILD), hypersensitivity pneumonitis, drug- or toxin-related ILD, post-infectious interstitial disorders, and idiopathic interstitial pneumonias, reflecting the heterogeneous pathogenic mechanisms underlying disease onset and progression [1]. Indeed, as observed in the available literature, multiple heterogeneous classification approaches have been proposed over time, underscoring substantial variability in how chILD entities are categorized. Current experience underscores the need for continued refinement and harmonization of classification systems, increasingly guided by genetic and molecular stratification, in order to better capture the expanding spectrum of underlying genetic mechanisms [2].

Furthermore, recent evidence highlights that ILDs may also develop in immunocompromised children, especially those with primary or secondary immunodeficiencies [4]. Respiratory involvement, particularly interstitial lung disease (ILD), represents a major cause of morbidity and mortality among patients with primary immunodeficiencies (PID) and, in some cases, may be the first clinical manifestation of the underlying immune disorder [5]. In this context, ILD can be regarded as a pulmonary expression of systemic immune dysregulation and may substantially influence long-term outcomes [6]. ILDs also represent one of the most significant immune-mediated complications of PID, with granulomatous-lymphocytic interstitial lung disease (GLILD) representing the most frequent presentation. GLILD is currently used as an umbrella definition that includes a heterogeneous spectrum of pulmonary lymphoid proliferations—such as lymphocytic interstitial pneumonia, follicular bronchiolitis, nodular lymphoid hyperplasia and granulomatous disease—as well as patterns consistent with organizing pneumonia [5]. In a multicenter cohort, ~40% of immunocompromised pediatric patients showed interstitial changes associated with underlying genetic or immunologic abnormalities [4].

In summary, pediatric interstitial lung diseases (chILD) represent a complex and evolving spectrum of disorders whose understanding has greatly advanced thanks to multidisciplinary collaboration and genetic research. At the same time, the extent to which genetic testing and multidisciplinary evaluation are incorporated into routine diagnostic pathways remains highly variable across centers. Key challenges include defining the role of genotype-driven classification, interpreting variants of uncertain significance, and integrating clinical, radiologic, and molecular data in settings with limited resources. Diagnostic overlap among developmental, immune-mediated, and environmental forms persists, underscoring the need for robust genetic and molecular approaches as well as international collaboration to harmonize definitions, improve diagnostic accuracy, and advance precision therapies and outcomes in pediatric ILDs. Ultimately, the integration of clinical, radiological, and genomic perspectives offers the most promising pathway toward precision medicine and improved long-term outcomes for children affected by ILDs.

### 1.2. Epidemiology and Clinical Relevance

The prevalence of chILD remains difficult to quantify and is likely underestimated due to inconsistent definitions, heterogeneous diagnostic criteria, and the absence of standardized reporting systems. Estimated prevalence reported in several studies vary widely, ranging from 0.1 to 16.2 cases per 100,000, depending on population, case definition, and study design. These numbers are difficult to ascertain and are likely underestimated [7]. Although ILD is rare, its incidence and prevalence appear to be increasing, likely due to improved epidemiological study methods, enhanced diagnostic resources and precision, and greater disease awareness [8].

Overall 5-year survival was 57.3% for children diagnosed before 2 years of age and 86% for those diagnosed between 2 and 18 years, confirming the poorer prognosis of congenital and early-onset forms. [7] The highest frequency is observed in younger patients, with a male predominance. Familial clustering occurs in approximately 10% of cases [2].

Large-scale registries and collaborative consortia such as chILD-EU, RespiRare, and ARNOLD have implemented standardized diagnostic protocols and epidemiological assessments. These networks have provided the first reliable prevalence estimates—approximately 1.5 cases per million children—and a mortality rate of 7% in immunocompetent pediatric populations. Contributions of cases to international registers are crucial to move beyond isolated case reports, which often lack generalizable insights [9].

Therefore, chILDs appear to be approximately ten times rarer than in adults [10]. Nevertheless, their presentation is often nonspecific and subtle, which can delay diagnosis and adversely affect prognosis. In this regard, the median age at symptom onset was 3 months, whereas the median age at diagnosis was 8.5 months, highlighting a clinically relevant diagnostic delay [2]. When ILD is suspected in a child, further investigations should be conducted within a multidisciplinary setting and interpreted by experienced radiologists, geneticists, and pathologists with specific expertise in chILD [10].

In recent years, most European countries have established dedicated specialists in expert centers and developed chILD referral networks. The Clinical Research Collaboration for chILD-EU, supported by the European Respiratory Society (ERS CRC chILD-EU), aims to improve chILD diagnosis and management through standardized approaches and collaborative research [10].

Regarding etiology, the most frequent causes in children under 2 years of age were surfactant metabolism disorders (16.3%) and NEHI (11.8%), whereas among children aged 2–18 years, the main causes were diffuse alveolar hemorrhage (12.2%), connective tissue disease–associated ILD (11.4%), hypersensitivity pneumonitis (8.8%), and sarcoidosis (8.8%) [11]. These multicenter data underscore that chILD may not be as rare as previously assumed, emphasizing the need for internationally harmonized registries and multicenter collaboration to improve diagnosis, treatment, and prognosis stratification.

However, available epidemiological data remain limited and heterogeneous, underscoring the need for robust international registries and collaborative networks to enhance disease characterization, diagnosis, and management.

### 1.3. Importance of Genetics in Pediatric ILDs

Genetic mechanisms play a crucial role in determining the onset, severity, and prognosis of pediatric interstitial lung diseases. Over the past two decades, advances in molecular genetics have identified genes involved in ILD pathogenesis, encompassing pathways related to surfactant metabolism, telomere maintenance, immune regulation, and epithelial repair [1]. Growing evidence indicates that both genetic and epigenetic factors contribute to ILD development, with genetic predisposition representing a major risk factor, particularly evident in idiopathic pulmonary fibrosis (IPF). Recent findings also highlight the role of epigenetic regulatory mechanisms—such as DNA methylation, histone modifications, or non-coding RNAs including microRNAs—in disease pathogenesis; however, a detailed discussion of epigenetic mechanisms is beyond the scope of the current review. Environmental and host-related factors further modulate disease expression: even in familial ILD with documented genetic causes, exposures such as viral infections, tobacco smoke, and airborne contaminants can influence disease onset, severity, and progression [2].

Beyond their etiological significance, genetic discoveries are profoundly reshaping diagnostic strategies in pediatric ILD. A recent analysis from the chILD-EU registry demonstrated that early genetic testing aimed at identifying the underlying cause of respiratory dysfunction in newborns can often obviate the need for video-assisted thoracoscopic surgery (VATS) biopsy, particularly in cases involving surfactant gene mutations or alveolocapillary dysplasia with misalignment of the pulmonary veins. Therefore, genetic testing should be considered as early as possible in the diagnostic work-up of chILD in neonates [12]. Consequently, genetic testing should be considered as early as possible in the diagnostic work-up of neonates with suspected chILD. In parallel, the incorporation of genetic data into multidisciplinary diagnostic discussions (MDD) has become crucial for accurate variant interpretation and for establishing meaningful genotype–phenotype correlations [13].

Next-generation sequencing approaches, especially trio-based whole-exome or whole-genome sequencing, significantly enhance the ability to identify disease-causing genes and uncover new variants. Although autosomal dominant conditions with incomplete penetrance complicate segregation analysis, as asymptomatic relatives may still carry pathogenic variants, genetically supported diagnoses remain highly valuable. In particular, obtaining a molecular diagnosis can guide timely clinical decisions and may obviate the need for invasive lung biopsy, especially in cases where lung transplantation or palliative strategies must be considered [14].

Ultimately, the growing role of genetics is redefining pediatric ILD as a model for precision medicine in rare respiratory diseases—where early molecular diagnosis can guide individualized care, optimize outcomes, and inform preventive strategies for affected families. Despite advances in diagnostic technologies, a major unmet need remains the lack of fully standardized, internationally accepted diagnostic guidelines. Existing frameworks—such as those proposed by the ATS, ERS, and the chILD-EU network—provide valuable structure, yet significant variability persists in how centers apply clinical, radiologic, genetic, and histopathologic criteria. Harmonization of diagnostic pathways is essential to improve comparability across cohorts and ensure equitable access to appropriate evaluation strategies [12]. Furthermore, as genetic testing becomes increasingly integrated into routine evaluation of suspected chILD, its psychological, ethical, and social implications in pediatric populations must be carefully considered. Genetic results may influence family dynamics, generate anxiety or uncertainty, and have implications for future reproductive decisions. Issues related to informed consent, return of incidental findings, data privacy, and potential stigmatization require structured counseling and ethical safeguards, particularly in children who cannot autonomously consent to testing [15].

Ethical frameworks emphasize that genetic analyses in pediatrics should be performed only when results are expected to provide direct clinical benefit, avoiding unnecessary testing that may expose families to psychosocial burdens without actionable outcomes.

Taken together, these considerations highlight the importance of developing standardized, ethically grounded diagnostic pathways that responsibly integrate genetic information while supporting families throughout the diagnostic process.

### 1.4. Rationale and Scope of the Narrative Review

The rationale of this review is to provide a critical overview of the current knowledge regarding genetic determinants of chILD, emphasizing how these insights are reshaping diagnostic algorithms, refining prognostic stratification, and informing individualized management strategies. This narrative review draws upon recent studies, systematic reviews, and expert consensus to outline the evolving role of genetics in defining disease classification, improving early recognition, and supporting family counseling. Ultimately, this synthesis aims to bridge the gap between molecular discovery and clinical application, fostering a precision-medicine approach that can improve outcomes and quality of care for affected children and their families.

## 2. Methods

We conducted a literature search in PubMed and EMBASE on 23 September 2025 using predefined search strings that included terms related to the population, disease, and genetic factors of interest. The search was restricted to articles published in English from 2015 onwards and focused on the pediatric population. The full search strategies and results are provided in Supplementary Materials. All retrieved records were imported into Rayyan software (https://www.rayyan.ai; accessed on 23 September 2025) [14] and independently screened by all authors to identify relevant studies. A total of 1319 records were retrieved; after removing 175 duplicates, 1144 studies were screened.

Consistent with the narrative review design, formal systematic inclusion and exclusion criteria were not applied. Instead, study selection and prioritization were guided by predefined conceptual relevance principles established by the author group before full-text review. Priority was given to studies demonstrating clear clinical relevance, including those linking genetic variants to disease onset, phenotypic variability, progression, complications, or treatment response in children. Greater interpretive weight was assigned to genetic associations that had been replicated in independent cohorts, supported by adequate sample sizes, and analyzed using appropriate statistical methodologies. Large-scale genomic studies, such as gene panels, exome or genome sequencing cohorts, and meta-analyses, were considered particularly informative.

Evidence was further prioritized when supported by functional or mechanistic data, including in vitro or in vivo experimental models, gene or protein expression analyses, and pathway studies that clarified the biological role of implicated genes in lung development, surfactant metabolism, telomere maintenance, immune regulation, or epithelial repair. Methodological robustness was also taken into account during evidence synthesis, including clarity of ILD phenotype definitions, rigor of genetic testing approaches, suitability of comparison groups, and consideration of potential confounding factors.

When conflicting or heterogeneous findings were encountered, interpretation considered differences in cohort size, statistical power, age of onset, ancestry, clinical phenotyping, and variant classification criteria, as well as the extent of independent replication and the presence of functional validation. Findings from larger, well-characterized cohorts or those supported by mechanistic evidence were generally considered more robust. Divergent results were not excluded; rather, they are discussed to highlight areas of uncertainty and ongoing investigation in the field.

As this review was narrative in design, study selection and evidence synthesis were guided by structured expert judgment rather than by a fully systematic review framework. We therefore aimed to enhance transparency by explicitly describing the search strategy and the conceptual criteria used to prioritize the literature discussed.

## 3. Genetic Basis of Pediatric ILDs

**Figure 1:**
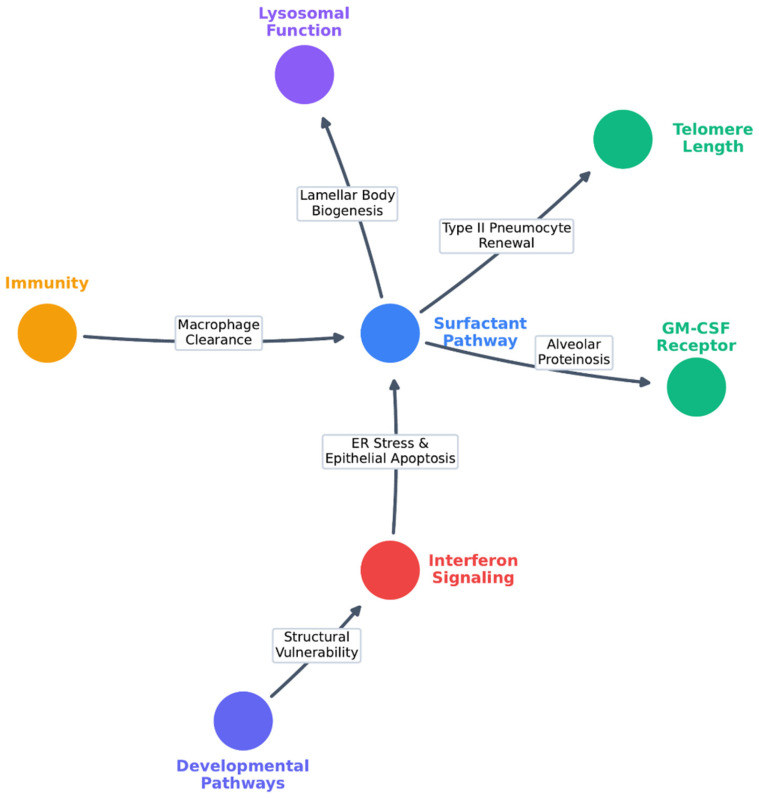
Integrative network of genetic pathways contributing to chILDs. Alterations in the surfactant pathway can lead to interalveolar deposits (proteinosis), particularly when GM-CSF receptor signaling is impaired. Lysosomal defects result in the accumulation of immature lamellar bodies, mimicking primary surfactant deficiencies. Chronic interferon signaling may induce ER stress and epithelial apoptosis, while telomere shortening limits type II pneumocyte renewal. Immune dysfunction further reduces macrophage-mediated clearance of surfactant and debris. Impaired alveolar capillary development can compromise lung architecture, potentially promoting early fibrosis. Overall, epithelial apoptosis combined with defective surfactant handling converge to disrupt pulmonary homeostasis.

Among chILD disorders, distinguishing between isolated and syndromic forms is essential. This classification provides clinicians with a practical approach to evaluating children with diffuse lung disease and determining whether additional systemic manifestations are present (Table 1). In parallel, it is important to emphasize that not all of the genes listed are unambiguously associated with chILDs. For some, the available evidence supports only a potential modifier effect, whereas for others the genotype–phenotype correlation remains under active investigation. Figure 1 summarizes the genetic pathways implicated in chILDs.

### 3.1. Isolated chILDs

These clinical conditions involve the lungs predominantly or exclusively. They typically affect the alveolar epithelium, surfactant metabolism, lipid processing, lysosomal function or alveolar macrophage biology [16].

#### 3.1.1. Genes Associated with Surfactant-Related Disorders

- *SFTPC* (OMIM *178620) encodes pulmonary-associated surfactant protein C (SPC) and is associated with *Surfactant metabolism dysfunction, pulmonary, 2* (OMIM #610913), an autosomal dominant disorder with an early onset in full-term newborns. SFTPC-related disease is most commonly inherited in an autosomal dominant manner, frequently due to de novo heterozygous variants, and is characterized by reduced penetrance and marked variable expressivity. Although neonatal presentation has been well described, *SFTPC* variants are associated with a broad clinical spectrum, ranging from severe neonatal respiratory distress to infantile- and childhood-onset ILD, and, more rarely, later-onset forms. Rare alternative or complex inheritance patterns, including autosomal recessive transmission, have been reported, but represent exceptional cases. Clinical severity may be exacerbated by recurrent respiratory infections. Affected individuals commonly exhibit failure to thrive, digital clubbing, hypoxemia, and respiratory distress with tachypnea or dyspnea. Thoracic CT scan typically reveals ground-glass opacities with interlobular septal thickening [17]. Histopathologic findings often include intra-alveolar accumulation of abnormal pro-SPC protein, type II pneumocyte hyperplasia and features of alveolar proteinosis [18,19,20]. Notably, a critical cysteine residue in the SFTPC BRICHOS domain has been identified; its mutation induces ER stress in vitro, suggesting a mechanistic link between specific variants and cellular dysfunction [18].
- *SFTPB* (OMIM *178640) encodes surfactant protein B (SPB) and is associated with *Surfactant metabolism dysfunction, pulmonary, 1* (OMIM #265120), an autosomal recessive disorder with phenotypic heterogeneity, characterized by neonatal onset and rapid clinical deterioration. Affected infants typically present with severe respiratory distress, tachypnea/dyspnea, pulmonary hypertension, and failure to thrive. Chest imaging commonly shows interstitial thickening and diffuse ground-glass opacities, while histopathology reveals alveolar proteinosis. A minimal or transient response to exogenous surfactant therapy may be observed, and death often occurs within the first months of life. Survival beyond infancy is exceedingly rare [21,22].
- *SFTPA1* (OMIM 178630) and *SFTPA2* (OMIM 178642) encode components of the lung surfactant protein A complex. Both genes are primarily associated with adult-onset interstitial lung disease, with pediatric cases reported only rarely and representing exceptional rather than typical chILD phenotypes. Their associated phenotypes, *Interstitial Lung Disease 1* (OMIM #619611) and *Interstitial Lung Disease 2* (OMIM #178500), show incomplete penetrance and marked variable expressivity. Importantly, disease penetrance is age-dependent rather than strictly limited to adulthood, even though most affected individuals develop symptoms later in life. For this reason, they are not primarily involved in chILD, although pediatric presentations have been reported on rare occasions. These disorders typically manifest with indolent interstitial pneumonia and recurrent respiratory infections; in some families, pulmonary fibrosis co-segregates with early-onset lung cancer [23].
- *ABCA3* (OMIM *601615) is expressed in alveolar type II pneumocytes and plays an essential role in surfactant synthesis. Pathogenic variants in this gene cause *Surfactant Metabolism Dysfunction, Pulmonary, 3* (OMIM #610921), a phenotypically heterogeneous autosomal recessive disorder. Although severe neonatal respiratory distress is a frequent presentation, *ABCA3* deficiency is now recognized as a disorder with a broad clinical spectrum, ranging from lethal neonatal respiratory failure to chronic infantile- and childhood-onset ILD. Biallelic loss-of-function variants usually result in lethal neonatal respiratory failure, whereas hypomorphic or missense variants may allow survival beyond infancy and lead to a chronic form of ILD. Chest CT generally shows ground-glass opacities and evolving fibrosis, and lung biopsy frequently reveals abnormal lamellar bodies within type II pneumocytes [24,25,26,27]. Recent studies analyzing lung tissue from infants and children with *ABCA3* deficiency further support marked genotype–phenotype variability and age-dependent molecular and cellular features [26].

#### 3.1.2. Genes Associated with GM-CSF Receptor–Related Disorders

- *CSF2RA* (OMIM *306250) and *CSF2RB* (OMIM *138981) encode, respectively, the ligand-binding alpha subunit and the non–ligand-binding, affinity-enhancing beta subunit of the granulocyte–macrophage colony-stimulating factor (GM-CSF) receptor. *CSF2RA*, located in the pseudoautosomal region (PAR) of the X chromosome [28] is associated with *Surfactant metabolism dysfunction, pulmonary, 4* (OMIM #300770), a congenital pseudoautosomal recessive disorder characterized by failure to thrive, tachypnea, restrictive ventilatory impairment, and patchy ground-glass opacities on chest CT [29]. Phenotypic variability has been reported, ranging from severe neonatal respiratory distress to milder, slowly progressive disease. *CSF2RB* is associated with *Surfactant metabolism dysfunction, pulmonary, 5* (OMIM #614370), a recessive condition that more commonly presents in adulthood, although pediatric cases have been reported. The clinical spectrum spans from chronic, indolent respiratory symptoms to acute or progressive respiratory failure, and disease severity appears comparable in males and females. Imaging may reveal diffuse or patchy ground-glass opacities, septal thickening or a “crazy paving” patter [30]. In both disorders, defective maturation of alveolar macrophages leads to impaired surfactant clearance and accumulation of surfactant-rich material within the alveoli.

#### 3.1.3. Genes Linked to Disorders of Epithelial Stress Response, Autophagy, and Lysosomal Function

- *MUC5B* (OMIM *600770) encodes a secreted gel-forming mucin expressed in the airways. The common promoter variant is strongly associated with *Susceptibility to idiopathic pulmonary fibrosis* (OMIM #178500). Although its contribution to childhood-onset interstitial lung disease is considered rare, it is well recognized as a genetic modifier in adult fibrotic lung disease [31]. It is important to emphasize that its specific functional impact in pediatric ILDs remains unclear, and current evidence is largely derived from observational studies.
- *GRN* (OMIM *138945) encodes progranulin, an autocrine growth factor involved in lysosomal function, cellular stress responses, and immune regulation. A recent pediatric case report described a homozygous loss-of-function variant associated with early-onset parenchymal lung disease, characterized by an alveolar proteinosis–like pattern and features of chronic pulmonary inflammation [32]. While this association is intriguing, these findings remain preliminary, and the functional impact of *GRN* variants in pediatric ILDs has yet to be fully elucidated.
- *HMOX1* (OMIM *141250) encodes heme oxygenase and has been implicated in susceptibility to *chronic obstructive pulmonary disease* (OMIM #606963). A recent case report described a young boy presenting with chronic respiratory failure due to nonspecific interstitial pneumonia, following recurrent infection-triggered hyperinflammatory episodes, including hemolysis [33]. Given that the available evidence is limited to a single reported case, current data remain insufficient to establish a definitive causal role for *HMOX1* variants in pediatric ILD.
- *LAMP3* (OMIM *605883) encodes lysosome-associated membrane glycoprotein 3, a protein involved in the regulation of lysosomal function and surfactant processing. Very recently, biallelic variants in LAMP3 have been associated with heterogeneous pediatric interstitial lung disease, including surfactant-related pulmonary manifestations [34]. Although these reports suggest potential pathogenic roles, further studies are required to determine whether *LAMP3* variants act as primary causative factors or as modifiers of disease severity.

Taken together, despite the genetic heterogeneity described above, isolated chILDs largely converge on a limited set of pathogenic mechanisms, primarily involving disrupted surfactant metabolism, alveolar epithelial stress, and impaired macrophage-mediated clearance. These shared mechanisms are reflected in a restricted spectrum of radiological patterns, most notably diffuse ground-glass opacities and pulmonary alveolar proteinosis–like features, particularly in early-onset disease. This convergence supports early genetic testing in children with unexplained ILD, as molecular diagnosis may clarify etiology, inform prognosis, and reduce the need for invasive diagnostic procedures. However, genetic findings should not be interpreted deterministically, as incomplete penetrance, variable expressivity, and the contribution of modifier genes substantially influence disease onset, severity, and clinical course. Consequently, molecular results must be integrated with clinical, radiological, and environmental data to guide longitudinal management and family counseling.

### 3.2. Syndromic chILDs

Across the syndromic forms discussed below, phenotypic variability, incomplete penetrance, and multisystem involvement represent shared and recurring features rather than syndrome-specific exceptions. These conditions result from defects in fundamental cellular pathways—including telomere maintenance, immune regulation, cytoskeletal organization, protein translation, and embryonic lung development—and manifest as multisystem disorders in which interstitial lung disease constitutes one component of a broader phenotype.

#### 3.2.1. Genes Associated with Telomere Maintenance Disorders

Telomere biology disorders provide a paradigmatic example of syndromic chILD, in which interstitial lung disease may represent an early or initial manifestation of a broader multisystem phenotype.

- *PARN* (OMIM *604212) encodes an exoribonuclease that shortens mRNA poly(A) tails through deadenylation, thereby regulating gene expression [35]. Pathogenic variants in *PARN* are associated with *Pulmonary fibrosis and/or bone marrow failure syndrome, telomere-related, 4* (OMIM #616371), an autosomal dominant condition characterized by variable expressivity, incomplete penetrance and typically adult onset. Affected individuals most commonly present with pulmonary fibrosis and may also exhibit premature graying of the hair. PARN-related features have also been reported in a child with ILD, who showed marked shortening of lymphocyte telomere length [36]. However, this association cannot be considered definitive, given the limited pediatric evidence and the currently unclear functional impact of these variants in early-onset disease.
- *TERC* (OMIM *602322) and *TERT* (OMIM *187270) encode essential components of the telomerase complex, the reverse transcriptase machinery responsible for adding repetitive DNA sequences to telomeres [37,38]. Pathogenic variants in *TERC* and *TERT* are associated, respectively, with *Pulmonary fibrosis and/or bone marrow failure syndrome, telomere-related, 2* (OMIM #614743) and *Pulmonary fibrosis and/or bone marrow failure syndrome, telomere-related, 1* (OMIM #614742). Both conditions follow an autosomal dominant inheritance pattern and demonstrate variable expressivity, incomplete penetrance, and typically adult onset. Clinical manifestations include pulmonary fibrosis, premature graying of the hair, bone marrow failure or pancytopenia, and an increased risk of cancer, leukemia, or myelodysplastic syndrome. Although pediatric cases have been described [39], the mechanisms by which specific variants contribute to early-onset ILD remain only partially understood, and their role as potential modifiers cannot be excluded.
- *TINF2* (OMIM *604319) encodes a core component of the 6-protein shelterin/telosome complex. Heterozygous pathogenic variants in *TINF2* are associated with *Dyskeratosis congenita, autosomal dominant 3* (OMIM #613990), a multisystemic condition characterized by variable onset from childhood to adulthood. Reported clinical manifestations include short stature, intrauterine growth restriction (IUGR), microcephaly, deafness, ocular abnormalities (such as nasolacrimal duct obstruction, epiphora, and retinopathy), oral leukoplakia, premature tooth loss, pulmonary fibrosis, respiratory insufficiency, enteropathy, portal hypertension, cryptorchidism, osteoporosis, reticular skin pigmentation, nail dystrophy, premature graying of the hair, alopecia, speech and learning difficulties, cerebellar hypoplasia or ataxia, bone marrow failure or pancytopenia, and an increased risk of cancer, leukemia, or myelodysplastic syndrome. Patients typically demonstrate marked telomere shortening and reduced telomerase activity, consistent with the broader spectrum of telomere-biology disorders [40,41]. While pulmonary fibrosis may occur, the severity and timing of lung involvement vary widely, underscoring the need for functional studies to clarify variant-specific effects.
- *NOP10* (OMIM *606471) encodes a protein component of the H/ACA small nucleolar ribonucleoprotein (snoRNP) complex. Pathogenic variants in NOP10 are associated with two distinct clinical entities: *Dyskeratosis congenita, autosomal recessive 1* (OMIM #224230), and an autosomal dominant form of *Pulmonary fibrosis and/or bone marrow failure syndrome, telomere-related, 9* (OMIM #620400). The autosomal recessive subtype (DKCB1) typically presents during adolescence and is characterized by nail dystrophy, skin hyperpigmentation, mucosal leukoplakia, neurodevelopmental disorders, bone marrow failure, lung and liver fibrosis, microcephaly and an increased risk of malignancies [42]. In contrast, the autosomal dominant form (PFBMFT9) generally exhibits adult onset, variable expressivity, incomplete penetrance and notably shows genetic anticipation [43]. The functional consequences of *NOP10* mutations in pediatric ILDs remain insufficiently defined and warrant further investigation.
- *POT1* (OMIM *606478) encodes a protein that binds the G-rich strand of telomeric DNA and plays a critical protective role in telomere maintenance. Pathogenic variants in POT1 are associated with an autosomal dominant form of *Pulmonary fibrosis and/or bone marrow failure syndrome, telomere-related, 8* (OMIM #620367). This condition typically shows adult onset, variable extrapulmonary manifestations, incomplete penetrance, and may exhibit genetic anticipation [44]. Although the association with lung disease is recognized, the contribution of *POT1* dysfunction to pediatric ILD is still being clarified.
- *DKC1* (OMIM *300126) encodes dyskerin, a core component of the telomerase complex and H/ACA ribonucleoprotein particles. Pathogenic variants in DKC1 cause *Dyskeratosis congenita, X-linked* (OMIM #305000), a multisystem disorder usually presenting from childhood to adolescence. Reported clinical features include short stature, intrauterine growth restriction (IUGR), microcephaly, ocular abnormalities, oral leukoplakia, premature tooth loss, pulmonary fibrosis, restrictive lung disease, gastrointestinal disturbances, hypospadias, phimosis or cryptorchidism, osteoporosis, reticular skin pigmentation, nail dystrophy, premature graying of the hair, neurodevelopmental abnormalities, cerebellar hypoplasia or ataxia, bone marrow failure, increased susceptibility to malignancies (including pancreatic and squamous cell carcinoma), and a heightened predisposition to chromosomal instability [45,46]. At present, the mechanisms linking *DKC1* dysfunction to lung involvement in pediatric patients remain insufficiently understood and require further functional and translational investigation.

Despite the heterogeneity of the genes discussed, telomere biology disorders share a common pathogenic mechanism involving impaired telomere maintenance, progressive telomere shortening, and tissue-specific vulnerability [47]. Phenotypic variability within and between families is influenced by multiple factors, including inherited baseline telomere length, residual telomerase activity, genetic anticipation, environmental exposures, and additional genetic modifiers [48]. In pediatric patients, telomere-related disorders may present with atypical, incomplete, or isolated features. Early-onset ILD can occur before the development of classic adult-onset manifestations such as pulmonary fibrosis or bone marrow failure [49]. Current evidence is largely based on genetic associations and telomere length measurements, which, while supportive, are insufficient to fully establish variant pathogenicity, particularly in early-onset disease [50]. Functional studies using patient-derived models remain limited, highlighting a gap in understanding genotype–phenotype correlations [51].

#### 3.2.2. Genes Implicated in Immune Function and Interferon Signaling

- *STING1/TMEM173* (OMIM *612374) encodes a transmembrane protein localized to the endoplasmic reticulum which plays a pivotal role in the production of type I interferons, particularly interferon β. Gain-of-function (GoF) mutations lead to constitutive activation of the pathway, causing severe autoinflammation in *STING-associated vasculopathy with onset in infancy* (SAVI, OMIM #615934), which is an autosomal dominant condition. Persistent IFN-β overproduction results in systemic vasculitis, which is often accompanied by telangiectasia, erythema or livedo reticularis, pulmonary fibrosis with alveolar macrophage infiltration and recurrent respiratory infections. Additional features include growth retardation, nail dystrophy, anaemia and various immunological abnormalities [52,53].
- *ISG15* (OMIM *147571) encodes a ubiquitin-like protein involved in ISGylation and regulation of type I interferon responses. Biallelic loss-of-function (LoF) variants impair negative feedback control, leading to excessive IFN activation. The resulting pediatric interferonopathy is characterized by interstitial pneumonia, lung fibrosis, systemic inflammation (including cutaneous lesions and basal ganglia calcifications), and poor viral control. Although functional data remain limited, the pathogenic mechanism is thought to reflect unrestrained IFN-mediated tissue injury [54].
- *COPA* (OMIM *601924) encodes a subunit of the coatomer protein complex I (COPI), which is essential for Golgi-to-ER retrograde transport. Pathogenic variants cause *Autoinflammation and autoimmunity, systemic, with immune dysregulation 1* (OMIM #616414), an autosomal dominant condition. Defective retrograde trafficking leads to ER stress and aberrant activation of type I interferon pathways. Pulmonary involvement is prominent and reflects immune-mediated alveolar injury, presenting as interstitial lung disease with ground-glass opacities, alveolar hemorrhage, and cystic remodeling. Pleiotropic manifestations may include hepatic, renal, and rheumatologic features. Clinical variability likely reflects differences in immune activation thresholds and modifying factors. Onset usually occurs during the first or second decade of life [55].
- *STAT5B* (OMIM *604260) encodes a transcription factor required for cytokine signaling and growth hormone (GH) pathway activity. Biallelic pathogenic variants cause *Growth hormone insensitivity with immune dysregulation 1* (OMIM #245590), an autosomal recessive early-onset disorder characterized by growth failure, impaired immune function, and recurrent respiratory infections. Pulmonary involvement likely reflects a combination of chronic inflammation, increased susceptibility to infections, and reduced epithelial regenerative capacity, which together may predispose to early-onset fibrotic ILD [56].
- *STAT3* (OMIM *102582) encodes a member of the STAT protein family involved in transcriptional regulation downstream of multiple cytokines. Monoallelic GoF variants are typically associated with *Autoimmune disease, multisystem, infantile-onset 1* (OMIM #615952), leading to constitutive *STAT3* pathway activation and loss of peripheral immune tolerance. Affected children commonly present with early-onset autoimmunity (including enteropathy, dermatitis, cytopenias, and type 1 diabetes), growth impairment, and susceptibility to recurrent infections. Lung involvement is thought to reflect chronic immune-mediated injury and impaired regulation of inflammatory responses, which may predispose to ILD in severe cases. Expressivity is highly variable, influenced by modifier genes and immune-maturation dynamics [57].
- *GATA2* (OMIM *137295) encodes a hematopoietic transcription factor essential for the development of monocytes, dendritic cells, and NK-cells. Monoallelic pathogenic variants cause *Immunodeficiency 21* (OMIM #614172), an autosomal dominant disorder with broad phenotypic heterogeneity. Pulmonary disease is frequent and includes alveolar proteinosis and progressive ILD, likely related to impaired macrophage function and defective surfactant clearance. The condition shows marked variability in penetrance and age at onset, ranging from childhood to adulthood, reflecting both environmental triggers and immune-system reserve [12,58].
- *OAS1* (OMIM *164350) encodes an interferon-induced enzyme involved in antiviral RNA degradation through the OAS–RNase L pathway. Heterozygous de novo GoF variants are associated with *Immunodeficiency 100 with pulmonary alveolar proteinosis and hypogammaglobulinemia* (OMIM #618042), a neonatal-onset condition with poor life expectancy, characterized by constitutive activation of the OAS pathway and excessive tissue injury. Affected patients typically present with respiratory insufficiency, lung consolidations, alveolar proteinosis, systemic inflammation (rash, gastrointestinal involvement), and severe immune dysfunction. The pathogenic mechanism likely reflects uncontrolled RNA-degradation signaling and impaired macrophage function, contributing to early and severe lung disease [12].
- *CCR2/MCP-1* (OMIM *601267) encodes a G-protein–coupled receptor for CCL2, playing a central role in monocyte recruitment and inflammatory trafficking. Biallelic pathogenic variants have been associated with a recessive form of *Polycystic lung disease* (OMIM #219600). Reported pediatric cases show progressive evolution with alveolar proteinosis and mild interstitial fibrosis, suggesting that impaired CCR2-mediated monocyte signaling disrupts alveolar homeostasis and macrophage-dependent surfactant clearance. Although data are limited, pleiotropic immune effects and marked clinical variability may complicate diagnosis and prognostication [59,60].
- *AP3B1* (OMIM *603401) encodes the large B1 subunit of the adaptor-related protein complex-3, which is required for vesicular trafficking in immune and epithelial cells. Biallelic pathogenic variants cause *Hermansky–Pudlak syndrome type 2* (OMIM #608233), characterized by neutropenia, recurrent infections and, in some reported cases, fibrosing ILD. The pulmonary phenotype likely results from defective lysosomal trafficking and impaired macrophage and neutrophil function, predisposing to chronic infection-driven inflammation. Expressivity is variable, and the specific contribution of AP3-complex dysfunction to lung pathology remains an active area of investigation [61,62].

#### 3.2.3. Genes Involved in Cytoskeletal Organization and Structural Integrity

- *FLNA* (OMIM *300017) encodes filamin A, a cytoskeletal crosslinking protein involved in mechanotransduction, cellular adhesion, and stabilization of epithelial architecture. Heterozygous variants have recently been associated with an X-linked form of interstitial lung disease, often accompanied by cardiac anomalies. Reported cases show marked variability in age at onset—from fetal life to young adulthood—suggesting incomplete penetrance and potential influence of modifier genes or environmental factors. Mechanistically, impaired cytoskeletal integrity may disrupt alveolar stability and mechanosensing, predisposing to early tissue remodeling [63,64].
- *ACTA2* (OMIM * 102620) encodes the smooth muscle α-actin isoform, which is essential for vascular and airway smooth-muscle contractility. Monoallelic de novo mutations are associated with *Smooth Muscle Dysfunction Syndrome* (OMIM #613834), characterized by congenital mydriasis, cerebrovascular anomalies, cardiovascular defects, gastrointestinal dysmotility, and ILD. Pulmonary disease may result from abnormal airway tone, dysregulated pulmonary vascular dynamics, and impaired tissue support, frequently presenting as tachypnea in infancy [65].
- *ITGA3* (OMIM *605025) encodes an integrin involved in cell–matrix adhesion within epithelial and renal tissues. Biallelic LoF variants are associated with *Epidermolysis Bullosa Junctional 7 with interstitial lung disease and nephrotic syndrome* (ILNEB, OMIM #614748), a severe multisystemic condition characterized by poor life expectancy, early respiratory distress, renal fibrosis, skin fragility, hypotonia, growth delay, and microcephaly. The pulmonary component likely reflects defective epithelial–basement membrane interactions and impaired tissue integrity [66].
- *ARHGAP42* (OMIM *615936) encodes a Rho GTPase–activating protein involved in cytoskeletal regulation and smooth-muscle tone, although a definitive clinical phenotype has not yet been established. Evidence linking *ARHGAP42* to ILD remains preliminary and is primarily derived from isolated case reports. One recently described patient carrying a homozygous stop-gain variant presented with ILD, systemic hypertension, and immune abnormalities, suggesting that *ARHGAP42* dysfunction may contribute to a broader syndromic spectrum [67]. However, given the limited number of reported cases and the absence of functional validation, current evidence remains insufficient to establish a definitive causal role for *ARHGAP42* variants in pediatric ILD.

#### 3.2.4. Genes Implicated in Embryonic Lung Development and Transcriptional Regulation

- *NKX2-1* (OMIM *600635) encodes a transcription factor essential for the early development of the thyroid, lung, and forebrain. Mutations in this gene are associated with *Choreoathetosis, hypothyroidism, and neonatal respiratory distress* (OMIM #610978), an autosomal dominant condition primarily characterized by congenital hypothyroidism, neurological manifestations (neonatal or early childhood hypotonia evolving into benign hereditary chorea) and pulmonary involvement, which may present as ILD, pulmonary fibrosis, or respiratory distress accompanied by tachypnea, cough, and recurrent airway infections. These manifestations likely reflect impaired transcriptional regulation during early lung patterning and epithelial–mesenchymal signaling, although the precise molecular mechanisms remain incompletely understood [68,69].
- *TBX4* (OMIM *601719) encodes transcription factor of the T-box family involved in embryonic development of the lungs and skeleton. Mutations in this gene are associated with *Ischiocoxopodopatellar syndrome, with or without pulmonary arterial hypertension* (OMIM #147891), which is characterized by short stature, cleft palate, osteoarticular abnormalities and idiopathic pulmonary hypertension. Over the past few years, the spectrum of TBX4-related pulmonary phenotypes has been expanded, including severe parenchymal lung disorders such as acinar dysplasia. Pulmonary manifestations likely result from altered transcriptional regulation during lung development, and disease severity and age at onset are variable, suggesting contributions from genetic modifiers and environmental factors [70,71].
- *FOXF1* (OMIM *601089) encodes Forkhead box protein F1, a member of the Forkhead transcription factor family. Monoallelic pathogenic variants are associated with *Alveolar capillary dysplasia with misalignment of pulmonary veins* (OMIM #265380), a neonatal-lethal disorder characterized by cardiovascular defects, gallbladder agenesis, annular pancreas, intestinal malrotation or atresia, urinary tract abnormalities, and severe early-onset pulmonary involvement with poor prognosis. Pulmonary pathology likely results from disrupted vascular–alveolar alignment and impaired alveolar maturation, reflecting altered transcriptional regulation during lung development, although detailed mechanistic studies remain limited [72,73,74].
- *FGF10* (OMIM *602115) encodes fibroblast growth factor 10, an essential regulator of pulmonary branching morphogenesis. Heterozygous pathogenic variants have been reported in children with early-onset ILD, presenting as postnatal respiratory failure. Histopathology from fatal cases reveal diffuse developmental lung alterations, (i.e., acinar dysplasia and interstitial fibrosis), with some patients also developing pulmonary hypertension. These findings suggest that impaired *FGF10* signaling disrupts epithelial proliferation and branching morphogenesis, although functional validation remains incomplete [75].

#### 3.2.5. Genes Involved in Protein Synthesis and tRNA-Charging Processes

- *FARSA* (OMIM *602918) and *FARSB* (OMIM *609690) encode the catalytic α- and β-subunits of cytoplasmic phenylalanine-tRNA synthetase (FARS1). Biallelic pathogenic cause *Rajab interstitial lung disease with brain calcifications 1 and 2* (OMIM #613658 and #619013), two multisystemic conditions with high variable expressivity. Clinical manifestations typically include interstitial lung disease, leukoencephalopathy with brain cysts and intracranial calcifications, microcephaly, hepatic dysfunction, hypoalbuminemia, skin and joint hyperlaxity, growth retardation, and distinctive dysmorphic features (elfin-like facies) [76].
- *MARS1* (OMIM *156560) encodes a cytoplasmic methionyl-tRNA synthetase, a member of a class I aminoacyl-tRNA synthetase. Biallelic pathogenic variants are associated with *Interstitial lung and liver disease* (OMIM #615486), typically presenting in infancy with interstitial lung disease characterized by pulmonary fibrosis and, in some cases, alveolar proteinosis, along with progressive liver dysfunction and failure to thrive. The clinical course is heterogeneous and may progress to respiratory failure [77,78].
- *YARS1* (OMIM *603623) encodes the cytoplasmic tyrosyl-tRNA synthetase. Biallelic pathogenic variants cause *Infantile-onset multisystem neurologic, endocrine, and pancreatic disease 2* (OMIM #619418), a condition with variable age at onset and clinical severity. Reported clinical features include neurodevelopmental impairment, poor prenatal and postnatal growth, gastrointestinal and hepatic involvement, chronic anemia, endocrine abnormalities, cystic or fibrotic interstitial lung disease, retinitis pigmentosa, and sensorineural hearing loss [79].
- Biallelic variants in *AARS1* (OMIM *601065), *LARS1* (OMIM *151350), and *IARS1* (OMIM *600709) have been associated with variable interstitial lung involvement, including pulmonary alveolar proteinosis in some cases. These disorders fall within the broader spectrum of recessive cytoplasmic aminoacyl-tRNA synthetase (aARS) deficiencies, which marked phenotypic heterogeneity. Among all cytoplasmic aARS genes, *MARS1* and *FARSA/FARSB* deficiencies appear to be more consistently linked to prominent, and in some cases severe, respiratory manifestations [80].

#### 3.2.6. Additional Genes Associated with Fibrosing Multisystem Syndromes Include

- *NHLRC2* (OMIM *618277) encodes a protein implicated in reactive oxygen species (ROS)-induced apoptosis. Biallelic pathogenic variants in this gene cause *FINCA syndrome* (FIbrosis, Neurodegeneration and Cerebral Angiomatosis—OMIM #618278), an infantile multisystem fibrosing disorder in which severe pulmonary fibrosis is a prominent manifestation [81].
- *FAM111B* (OMIM *615584) encodes a trypsin-like cysteine/serine protease. Heterozygous mutations in this gene underlie hereditary fibrosing poikiloderma (OMIM #615704) with tendon contractures and may be associated with progressive pulmonary fibrosis in a subset of patients [82].

#### 3.2.7. Copy Number Variations (CNVs)

Two additional deletion syndromes are relevant in the context of childhood-onset interstitial lung disease:

- Deletion 5q (OMIM #153550) is commonly observed in myelodysplastic syndromes and has been reported in cohorts with associated interstitial lung disease; such chromosomal abnormalities should be considered hematologic predisposition factors for pulmonary complications [83].
- Contiguous *ABCD1*/*DXS1357E* deletion syndrome (CADDS, OMIM #300475) is a neonatal contiguous-gene deletion condition with multisystem involvement (including liver and nervous system) and occasional respiratory manifestations; it is best categorized among contiguous-gene syndromes with secondary pulmonary disease [84].

Overall, the syndromic forms of chILD discussed above arise from defects in fundamental cellular pathways, including telomere maintenance, immune regulation, cytoskeletal organization, protein synthesis, and lung development, resulting in multisystem disorders with pulmonary involvement. In pediatric patients, ILD may represent the initial or predominant manifestation, preceding overt extra-pulmonary features and complicating early recognition. Establishing a syndromic genetic diagnosis has important clinical implications, as it enables targeted surveillance for systemic complications and supports more accurate prognostic stratification. Nevertheless, the marked variability in expressivity and age of onset limits genotype–phenotype prediction and underscores the need for a multidisciplinary diagnostic and management approach.

**Figure 2:**
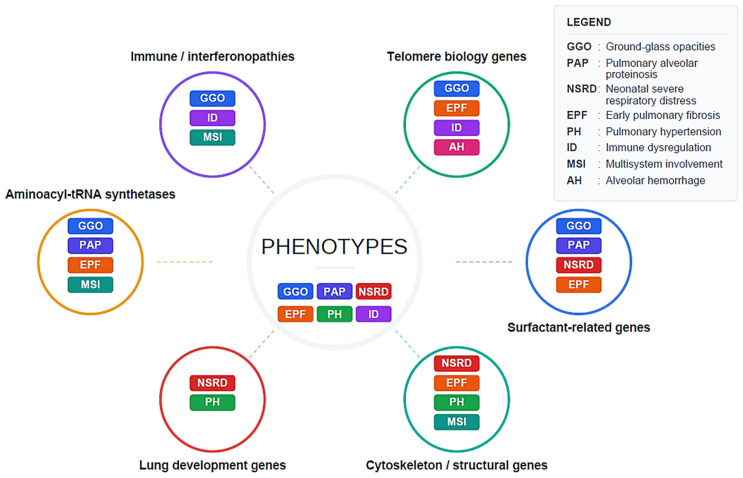
Genotype–phenotype correlations in chlLDs. Schematic representation of the main genetic categories implicated in chlLDs and their overlapping clinical and radiological phenotypes. Distinct gene groups—including surfactant-related genes, telomere biology genes, immune/interferonopathy-related genes, aminoacyl-tRNA synthetases, lung development genes, and cytoskeleton/structural genes—are associated with a partially shared spectrum of pulmonary manifestations such as ground-glass opacities (GGO), pulmonary alveolar proteinosis (PAP)-like patterns, neonatal severe respiratory distress (NSRD), early-onset pulmonary fibrosis (EPF), pulmonary hypertension (PH), and immune dysregulation (ID). The convergence of phenotypes across different molecular pathways highlights the complexity of genotype–phenotype relationships in chlLDs and underscores the need for integrated clinical, radiological, and genetic interpretation.

In summary, while the distinction between isolated and syndromic forms of chILD remains clinically useful as an organizing framework, it does not fully capture the biological continuum underlying many pediatric ILDs. As highlighted by the integrative overview in Figure 2, lung disease may represent either an apparently isolated phenotype or the earliest manifestation of a broader multisystem disorder. This conceptual overlap challenges rigid classification schemes and reinforces the need for integrated clinical and molecular interpretation when approaching pediatric ILD diagnosis, classification, and long-term management.

## 4. Diagnostic Clues

### 4.1. Typical Clinical Presentation in Infancy and Childhood

The clinical manifestations of pediatric ILD are often subtle and non-specific. Symptom onset is typically insidious, and many children may experience respiratory complaints for years before a definitive diagnosis is established. Presentations range from asymptomatic cases with incidental radiologic abnormalities suggestive of ILD to more characteristic findings marked by respiratory symptoms and recurrent exacerbations [7].

Children with ILD typically present with cough and tachypnoea, or may exhibit unexpectedly severe or persistent respiratory morbidity in the neonatal period or later after an acute viral respiratory infection. Failure to thrive is particularly common in infants and young children. Older children may present with chronic, slowly progressive dyspnea either at rest or with exercise intolerance [7,14].

According to Nayir Buyuksahin and Kiper [85], children who have at least 3 of the following 4 criteria for a minimum of 4 weeks should undergo evaluation for ILD: (1) respiratory symptoms/signs (such as cough, tachypnea, or dyspnea at rest or with exercise, crackles, retractions, digital clubbing, failure to thrive, or respiratory failure); (2) systemic arterial hypoxemia; (3) diffuse radiological abnormalities; (4) abnormal pulmonary function tests.

It is important to emphasize that the timing and severity of clinical presentation can provide valuable diagnostic clues. Severe respiratory distress in the immediate postnatal period often suggests developmental lung disorders or surfactant dysfunction diseases, which are typically associated with high morbidity and limited therapeutic options. In contrast, entities such as pulmonary interstitial glycogenosis (PIG) and NEHI usually present with milder symptoms, although they may still require prolonged supportive care and close follow-up [86].

Cough is a frequent symptom in interstitial lung diseases, with approximately 30–50% of patients with IPF in the general population reporting a cough severe enough to impair quality of life. Dyspnea is the hallmark of ILD, and typically first manifests during strenuous exertion. As disease progresses, patients experience gradual limitations in exercise capacity, and resting hypoxemia becomes common in advanced stages. The onset and rate of progression of dyspnea vary across different ILDs. Fatigue and unintentional weight loss may also develop over time, with weight loss serving as an adverse prognostic indicator. Digital clubbing is present in approximately 7–42% of individuals with pulmonary fibrosis. On lung auscultation, fine Velcro-like crackles at the bases are identified in 93% of patients with IPF and in 73% of those with non-IPF ILDs in the general ILD population, while individuals with hypersensitivity pneumonitis may exhibit high-pitched end-inspiratory squawks [1]. Wheezing may also be observed, although up to one-third of children may have normal auscultation. Additional clinical signs include subcostal retractions, chest wall deformity such as pectus excavatum, seen in some surfactant disorders, or pectus carinatum (more typical in airway disorders associated with air-trapping such as NEHI and bronchiolitis obliterans), and hypoxemia. Children may also show clinical features of pulmonary hypertension [14].

### 4.2. Challenges in Diagnosis and Differential Diagnosis

Given that the prevalence of individual entities is likely <1 per 100,000 individuals, most hospitals encounter so few cases that experience with diagnosis and management is often limited. Helpful guidelines outlining clinical assessment, imaging and/or genetic as well as histopathological analyses have been established to support accurate diagnosis [12]. In this section, we summarize the most recent evidence regarding the diagnostic process, focusing on the integration of clinical, radiological, genetic, and histopathological findings that have refined the current approach to the evaluation and classification of chILD.

Before pursuing a specific chILD diagnosis, more common causes of interstitial lung disease in children must be excluded. These include cystic fibrosis, acquired or congenital immunodeficiency, congenital heart disease, bronchopulmonary dysplasia, pulmonary infection, primary ciliary dyskinesia and recurrent aspiration. Clinical evaluation should include a thorough medical history—covering family history, prenatal course, and neonatal events—assessment of oxygen saturation at rest and during exercise, chest imaging (radiography and HRCT), laboratory testing with emphasis on immune function and autoantibodies, echocardiography, and pulmonary function testing (in children aged >4–5 years). Useful clinical protocols are available on the chILD-EU website (www.childeu.net). Echocardiography is essential for excluding structural cardiovascular disease and pulmonary hypertension (PH), which may occur in up to 7% of children evaluated for suspected ILD [12].

**Figure 3:**
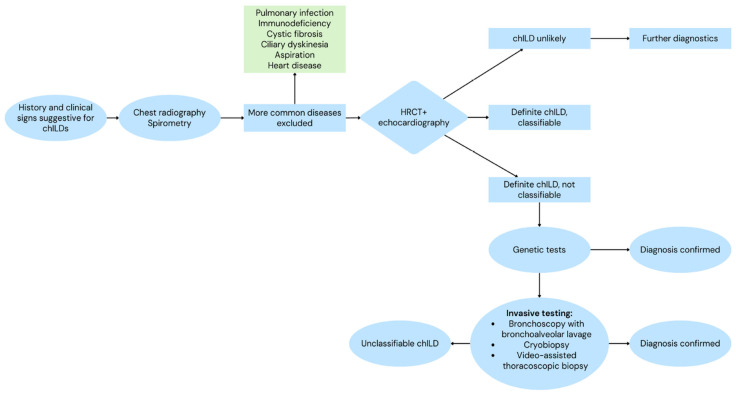
Diagnostic workflow for chILD. After clinical assessment and initial tests, common diseases are excluded. High-resolution computed tomography (HRCT) and echocardiography guide classification into definite or unlikely chILD. Genetic testing and, if required, invasive procedures confirm the diagnosis in unclassifiable cases.

Routine laboratory investigations (including complete blood count, serum markers for renal, hepatic, and thyroid function, and quantitative immunoglobulins) are used to screen for systemic or multiorgan disease. Serological testing for autoantibodies is particularly important in older children and in those with evidence of pulmonary hemorrhage, renal involvement, and articular, cutaneous, or systemic manifestations. IgG precipitin testing to allergens may assist in the evaluation of suspected hypersensitivity pneumonitis, though sensitivity and specificity are incomplete (i.e., a hypersensitivity to birds). Overall, a stepwise diagnostic approach is recommended, beginning with non-invasive testing (pulmonary function tests, imaging, echocardiography, and genetic testing) and followed, when indicated, by invasive investigations such as bronchoscopy and lung biopsy (Figure 3).

Oxygen saturation measured via pulse oximetry (SpO_2_) reflects pulmonary impairment and is a key component of chILD severity scoring systems. Standardization is essential to ensure clinical reliability. The chILD-EU group recommends measuring SpO_2_ in room air, with the child resting for 5 min and accepting a desaturation nadir of 80% before resuming supplemental oxygen if required. SpO_2_ measurement during sleep or dynamic testing (e.g., exercise in older children) may reveal abnormalities not evident at rest. However, inter-center variability in measurement protocols limits comparability across institutions [14]. When ILD is suspected or confirmed, pulmonary function testing, including forced vital capacity (FVC) and diffusing capacity for carbon monoxide (DLCO), should be obtained to assess disease severity, as ILD typically produces a restrictive ventilatory pattern [1].

The 2013 ATS guidelines propose three diagnostic algorithms, two for infants presenting with severe respiratory disease in the neonatal period (differentiated by the presence or absence of a family history of ILD) and one for children presenting after 1 month of age. In all three algorithms, imaging appears early in the diagnostic pathway, once common non-chILD conditions have been excluded [87]. Even when imaging does not yield a specific chILD diagnosis, it remains a key component of multidisciplinary evaluation. Many diffuse lung diseases demonstrate non-homogeneous lung involvement with geographic areas of sparing or variable disease patterns. Consequently, pre-biopsy imaging is essential to guide the surgeon toward an appropriately affected region of lung tissue and to support the subsequent interpretation of biopsy findings.

Genetic testing has become an increasingly important element of the chILD diagnostic work-up, especially in infants and even more so when there is a strong family history [88]. Genetic alterations have been reported in association with specific chILD entities in up to 20% of cases. In a recent analysis of the chILD-EU register, 46% of enrolled patients underwent genetic testing, and 13% of these achieved a final molecular diagnosis [12]. The overarching aim of both imaging and genetic studies is to establish the most specific diagnosis possible without resorting to invasive procedures, with diagnostic algorithms converging on surgical lung biopsy only when no alternative etiology can be identified [88]. Thus, the indication for genetic testing should be considered as early as possible during the diagnostic evaluation of neonates with suspected chILD [12].

Multidisciplinary discussion (MDD) represents a cornerstone of ILD diagnosis, allowing the integration of clinical, radiological, genetic, and pathological findings into a unified interpretative framework. Evidence from adult and pediatric ILD cohorts demonstrates that MDD significantly improves diagnostic accuracy and reduces interobserver variability, particularly in genetically mediated or radiologically ambiguous cases. As highlighted by Borie et al. (2019) [13], dedicated multidisciplinary teams markedly enhance variant interpretation and diagnostic confidence in inherited pulmonary fibrosis, underscoring the need for broader dissemination of such models and for improved referral pathways in pediatric ILD care. However, despite its recognized value, access to specialized ILD centers remains uneven, and limited availability of expert radiologists, geneticists, and pediatric pulmonologists can delay diagnostic confirmation, especially in rare or atypical presentations [13]. Strengthening referral pathways, promoting inter-center collaboration, and implementing virtual MDD models may help mitigate these disparities and ensure more equitable access to expert assessment.

Differentiating between pediatric ILD subtypes is challenging because many entities share overlapping clinical, radiologic, and sometimes histologic features. However, specific distinguishing clues can support a more accurate differential diagnosis. NEHI and PIG, for example, may both present in early infancy with tachypnea and hypoxemia, yet their imaging profiles differ: NEHI typically shows ground-glass opacification in the right middle lobe and lingula with associated air-trapping, whereas PIG demonstrates more diffuse ground-glass attenuation and septal thickening. Histologically, NEHI is characterized by an increased number of bombesin-positive neuroendocrine cells, while PIG features glycogen-rich interstitial mesenchymal cells. Surfactant dysfunction disorders (*SFTPB*, *SFTPC*, *ABCA3*, *NKX2-1*) may mimic chronic pneumonitis of infancy (CPI) or nonspecific interstitial pneumonia (NSIP) on imaging, with shared findings such as diffuse or patchy ground-glass opacities. Their distinction relies on a combination of genetic testing, age of onset, and specific radiologic and histologic patterns: *ABCA3* and *SFTPB* variants are frequently associated with pulmonary alveolar proteinosis (PAP), whereas *SFTPC* and *ABCA3* variants can be associated with CPI and desquamative interstitial pneumonia (DIP). “Crazy-paving” is more typical of SFTPB or ABCA3-related pulmonary alveolar proteinosis, while *SFTPC* variants often produce a combination of ground-glass and cystic changes [12]. Developmental lung disorders, including congenital alveolar dysplasia (CAD) and acinar dysplasia (AD), occur in term infants who present with immediate and severe respiratory failure, while prematurity-associated lung disease affects preterm infants and shows more variable clinical evolution. Histologically, AD is characterized by a complete absence of acini and alveoli, whereas CAD demonstrates late canalicular to early saccular architecture features that are not observed in acquired neonatal lung injury [12]. Alveolocapillary dysplasia with misalignment of pulmonary veins (ACD/MPV) can initially resemble persistent pulmonary hypertension of the newborn or severe neonatal ILD. The distinguishing diagnostic features—central capillary malposition, peribronchial venous dilation, and abnormal lymphatics—require histopathologic evaluation. Clinical characteristics, including profound hypoxemia refractory to ventilation, also help differentiate ACD/MPV from other neonatal ILDs. Hypersensitivity pneumonitis (HP) may overlap radiographically with NSIP or early-onset autoimmune ILD. Diagnostic differentiation relies heavily on exposure history, the presence of serum precipitins [89], mosaic attenuation with air-trapping, and bronchoalveolar lavage lymphocytosis [90]. Autoimmune or immunodeficiency-related ILD, including GLILD, may present with ground-glass opacities and lymphocytic infiltrates similar to LIP or NSIP. However, GLILD is typically accompanied by splenomegaly, lymphadenopathy, cytopenias, and systemic immune dysregulation [6], whereas isolated LIP/NSIP forms may occur without systemic involvement. In summary, differentiation between ILD subtypes relies on integrating clinical age-of-onset patterns, HRCT distribution abnormalities, histopathologic signatures, genetic findings, and systemic manifestations.

### 4.3. Radiography

Chest radiography remains the most widely used respiratory imaging tool globally, offering a rapid overview of the entire thorax in a single acquisition. However, in both children and adults, its sensitivity for detecting ILD is significantly lower than that of CT. Normal chest radiographs at initial presentation have been reported in approximately 10% to 42% of children who were later confirmed to have ILD. In clinical practice, it is uncommon to proceed to CT for suspected chILD without first obtaining a chest radiograph for baseline comparison. Despite its lower resolution, a high-quality chest radiograph provides a readily available, low-radiation option for longitudinal monitoring, avoiding the cumulative exposure associated with repeated CT examinations. For these reasons, chest radiography should be reviewed prior to initial CT assessment and should be considered the first-line modality for follow-up imaging in clinically stable children with ILD.

Given these considerations, the roles of chest radiography in chILD assessment include (1) raising the initial suspicion of ILD, (2) primary evaluation of disease distribution and appearance before CT imaging is available, (3) investigation of extrapulmonary features of specific chILD entities, (4) detecting signs of chronic aspiration (as a primary or exacerbating pathologic condition), and (5) as a follow-up evaluation imaging modality (ideally as part of a routine assessment, similar to the annual evaluations performed in cystic fibrosis care) [88].

Although chest radiography rarely yields a definitive diagnosis of chILD, it may identify conditions that mimic ILD (especially infection), and helps to define the extent and pattern of structural lung alterations [12].

### 4.4. Computed Tomography

Thoracic CT represents the primary diagnostic modality for identifying and characterizing ILD; in adult populations, it demonstrates approximately 91% sensitivity and 71% specificity for differentiating ILD subtypes. Distinct forms of ILD often present with characteristic CT patterns that correlate with underlying histopathology. However, neither CT imaging nor histology alone is sufficient to establish a definitive diagnosis [1]. The most common high-resolution CT (HRCT) feature of ILD is diffuse ground-glass attenuation, while intralobular lines, irregular interlobular septal thickening and honeycombing are observed less commonly. In some young children, large subpleural air cysts in the upper lobes adjacent to areas of ground-glass opacities—interpreted as paraseptal or irregular emphysema—have also been reported [7].

HRCT should be performed in specialized pediatric radiology centers to ensure diagnostic-quality imaging. HRCT shows a strong correlation with histology and has therefore become the reference standard for radiologic assessment. To optimize spatial resolution, thin-section acquisition, minimal field of view, and sharp reconstruction algorithms are generally recommended [7]. CT imaging informs the subsequent diagnostic approach by classifying findings into three categories: (1) chILD present, reliably classifiable, (2) chILD present, but not reliably classifiable, and (3) chILD unlikely [12]. CT accurately identifies and differentiates diffuse, nodular, cystic, bronchiectatic, and atelectatic or consolidative patterns. However, image quality (and interpretability) can be influenced by multiple factors, including scanner manufacturer, protocol settings, and patient preparation (i.e., spontaneous breathing, mechanical ventilation, or pressure-gated techniques). Use of phantoms and protocols across sites can help reduce variability for some of these factors. Nevertheless, the rapid technological advancements allowing fast scanning without anesthesia in young children introduce additional complexity and variability. A validated CT scoring system for chILD is currently lacking [14].

Certain chILD entities show relatively characteristic HRCT appearances. Hypersensitivity pneumonitis typically progresses from ill-defined centrilobular nodularity and ground-glass opacification in the acute phase, to better-defined centrilobular nodules in the subacute phase, and ultimately to coarse interlobular septal thickening, architectural distortion, and variable ground-glass attenuation in the chronic phase, often with areas of lobular hypoattenuation [88]. The CT appearance of NEHI is reportedly 100% specific. Patterns of diffuse ground glass opacity and reticulation are reported as common in infants and young children with surfactant genetic disorders [14]. The extent of ground-glass opacification correlates with clinical phenotype, with significantly more ground glass opacification in NEHI patients requiring continuous oxygen compared to those needing only nocturnal oxygen. While specificity is high, the sensitivity reported in the original study was 78% [88]. The typical HRCT features of pulmonary interstitial glycogenosis (PIG) include diffuse ground-glass opacities, interlobular septal thickening, and occasional small cystic changes, reflecting the cellular, non-inflammatory nature of the disease and its predominant interstitial involvement [88].

Imaging findings of lipoid pneumonia are variable, but include extensive, mass-like consolidation, sometimes with internal areas of low attenuation on CT [88].

If less invasive investigations, including CT imaging, fail to identify the underlying cause and clinical severity warrants, lung biopsy may be indicated to achieve a definitive diagnosis [14].

### 4.5. Histology

Histology has historically been considered the gold standard for diagnosis of chILD and forms the basis of all key classification systems. However, the increasing role of genetic testing is gradually reshaping this paradigm. Although lung biopsy can provide a diagnosis in most cases, it carries important limitations and inherent risks [14]. Surgical lung biopsy is associated with a mortality rate of 1% to 2%. Samples are obtained invasively, cannot be easily repeated, and may not adequately represent diseased regions of the lung. In infants and young children, an open or video-assisted thoracoscopic (VATS) biopsy is usually required, as transbronchial biopsy is generally insufficient. Proper tissue processing is critical, and with relatively few global histopathology experts for these rare conditions, inter-observer diagnostic variability remains a concern [14].

Therefore, although lung biopsy remains a key diagnostic tool in unresolved or clinically complex cases, its invasive nature and associated risks strongly underscore the need to develop and implement less invasive diagnostic alternatives, particularly in pediatric populations, in whom the available evidence remains limited.

Currently, fewer than 10% of patients with ILD undergo lung biopsy. In adult populations, in many centers, bronchoscopic transbronchial cryobiopsy—a minimally invasive procedure involving rapid freezing of lung tissue—has replaced VATS biopsy for obtaining tissue samples. Cryobiopsy offers a lower complication rate while providing similar diagnostic accuracy [1]. However, experience with cryobiopsy in pediatric ILD remains limited, and standardized protocols are lacking. Histopathologic characteristics are not unique to a specific ILD, and overlap exists among different histopathologic and radiologic findings. This is particularly evident in the four most common histopathologic patterns: usual interstitial pneumonia (UIP), nonspecific interstitial pneumonia (NSIP), organizing pneumonia, and diffuse alveolar damage [1].

Regarding the morphological patterns of chILD, the main microscopic and structural findings and their diagnostic implications are summarized below, based on Cunningham et al. and Laenger et al. [12,14]. This synthesis provides a practical overview of the histopathological characterization of these entities:

- Neuroendocrine cell hyperplasia of infancy (NEHI): inconspicuous architecture of septa, interstitium, and vessels, with an increased number of neuroendocrine cells in ≥70% of bronchi, often confirmed by bombesin-positive immunostaining, typically in otherwise normal pulmonary parenchyma.
- Developmental disorders (CAD/AD): pattern resembling canalicular or saccular lung development. Congenital alveolar dysplasia (CAD) shows enlarged, heavy lung with diffuse alveolar simplification, widened septa, reduced capillary density, and predominance of type II pneumocytes. Acinar dysplasia (AD) reflects a more severe maturation arrest at the pseudo-glandular or early canalicular phase, with a complete absence of acini and alveoli.
- Chronic neonatal lung disease, chromosomal abnormalities, or pulmonary hypoplasia: enlarged alveoli with reduced in numbers without increased cellularity.
- Pulmonary interstitial glycogenosis (PIG): a non-inflammatory interstitial disorder of infancy; diffuse or focal widening of alveolar septa with PAS-positive, glycogen-rich ovoid cells, preserving the alveolar epithelium.
- Alveolocapillary dysplasia with misalignment of pulmonary veins (ACD/MPV): typically presented with severe neonatal respiratory distress; centrally located septal capillaries, distended peribronchial veins, small arterial media hyperplasia, and lymphatic ectasia.
- Chronic pneumonitis of infancy (CPI): type II pneumocyte hyperplasia with interstitial edema and focal lymphoid infiltrates; similar patterns may also be observed in surfactant dysfunction, viral infections, or immunodeficiency.
- Lymphocytic interstitial pneumonia (LIP): dense lymphocytic infiltrate with fibrosis and lymphoid aggregates, often associated with autoimmune diseases or immunodeficiency syndromes.
- Nonspecific interstitial pneumonia (NSIP): less dense inflammatory infiltrates with fibrosis and septal thickening, potentially associated with surfactant dysfunction, autoimmune disease, or hypersensitivity pneumonitis.
- Pulmonary alveolar proteinosis (PAP): intra-alveolar accumulation of eosinophilic, granular, cell-poor material containing cholesterol clefts and foamy macrophages, with type II pneumocyte hyperplasia and an otherwise unremarkable interstitium.
- Desquamative interstitial pneumonia (DIP): alveoli filled with foamy macrophages, often linked to surfactant dysfunction, drug reactions, or toxic inhalation.
- Obliterative bronchiolitis: fibrous remodeling and obliteration of distal airways; may result from post-infectious injury, chronic lung allograft dysfunction, or graft-versus-host disease.
- Follicular bronchiolitis/bronchitis: nodular lymphoid infiltrates within bronchiolar walls, often seen in autoimmune disease or common variable immunodeficiency.
- Granulomatous inflammation: variably distribution, with or without necrosis, suggests infection, sarcoidosis, hypersensitivity pneumonitis, vasculitis, or immunodeficiency.
- Surfactant dysfunction disorders: type II pneumocyte hyperplasia with PAS-D–positive intra-alveolar material, intra-alveolar macrophages, septal fibrosis, and abnormal lamellar bodies, often associated with *SFTPB*, *SFTPC*, or *ABCA3* mutations.

### 4.6. MR Imaging

Although MR imaging has traditionally been limited by low signal intensity, high levels of susceptibility artifact at air-tissue interfaces, and the frequent need for general anesthesia, substantial advances in pulmonary MRI have been achieved in recent years [88]. MRI is beginning to gain a role alongside CT in thoracic imaging, offering particular value in the follow-up of selected, established diagnoses rather than serving as a primary diagnostic modality for newly suspected child [14]. However, at present, lung MRI remains largely investigational in this setting, and standardized protocols specific to pediatric ILD are still lacking.

### 4.7. Ultrasonography

Ultrasonography is increasingly being adopted as an initial imaging tool for lung assessment, including in adults with idiopathic interstitial lung disease and connective tissue disorder [14]. Although this approach may offer advantages in resource-limited settings, lung ultrasound lacks the ability to differentiate ILD from other conditions that similarly alter the interstitium (e.g., pulmonary edema or subsegmental atelectasis). Consequently, it is not an appropriate imaging modality for evaluating suspected chILD [88], and, like MRI, remains largely exploratory in this context.

### 4.8. Bronchoalveolar Lavage (BAL)

The diagnostic value of BAL in chILD has been examined in numerous studies with inconsistent correlation of the findings with specific chILD entities. Its principal value lies in excluding active infection and providing material for microbiological testing. When BAL is performed, samples should undergo standardized morphological assessment, including staining with hematoxylin–eosin, periodic acid–Schiff (PAS), iron, and Sudan. Therefore, although the diagnostic yield of BAL may be limited, if a bronchoscopy is performed, it should be included in the diagnostic work-up [12].

In any case, as reported by Maher [1], the diagnostic process for interstitial lung diseases should always be based on an integrated, multidisciplinary strategy. The accepted approach to ILD diagnosis is multidisciplinary assessment with a team consisting of pulmonologists, radiologists, and, where necessary, pathologists and rheumatologists. This approach emphasizes the need to synthesize clinical features with imaging data and, when indicated, histologic information. Nathan and colleagues [7] underscore the importance of a structured, stepwise diagnostic process, beginning with a detailed clinical assessment and advancing to imaging, molecular testing, and histopathological evaluation as necessary. The diagnostic pathway should always be guided by patient age, clinical presentation, and disease evolution, with the aim of identifying developmental, inflammatory, or genetic mechanisms underlying the disease process.

Notably, clinical presentation, radiologic patterns, histopathologic findings and genetic abnormalities not only support accurate diagnosis but also inform prognosis and therapeutic decision-making. For example, specific genetic mutations may guide the use of immunomodulatory therapies or determine eligibility for targeted pharmacological treatments, whereas the extent of parenchymal involvement on HRCT may indicate the need for supplemental oxygen or respiratory physiotherapy. Moreover, patients with progressive respiratory impairment or high-risk genotypes may require early discussion regarding referral pathways for lung transplantation.

## 5. Genetic Diagnosis

### 5.1. Genetic Testing Methodologies

The diagnostic workup for monogenic forms of interstitial lung disease increasingly relies on next-generation sequencing (NGS). Causative variants can be identified using targeted gene panels, whole-exome sequencing (WES), or whole-genome sequencing (WGS). The choice among these approaches is guided by the patient’s clinical presentation, the suspected biological pathway, and the need to detect specific variant types such as deep intronic or structural variants, which are more readily captured by WGS. At the same time, it is important to acknowledge the intrinsic limitations of genetic testing within the diagnostic pathway, including the possibility of incomplete coverage, reduced performance in GC-rich or repetitive regions, and limited sensitivity for detecting low-level mosaicism. To complement sequencing-based diagnostics, chromosomal microarray (CMA) analysis remains useful for detecting pathogenic CNVs that may underlie or contribute to the pulmonary phenotype. This is particularly relevant for identifying recurrent large deletions involving *CSF2RA* [91], as well as for detecting 5q deletion syndromes or contiguous-gene deletions associated with congenital alveolar dysplasia (CADDS). When somatic mosaicism or low-level clones are a concern (for example in hematologic disease), fluorescence in situ hybridization (FISH) on the appropriate tissue may be complementary.

In the context of suspected telomeropathies, NGS can identify pathogenic variants in genes involved in telomere maintenance (such as *TERT*, *PARN*, *RTEL1* or *DKC1*). However, genetic findings should be integrated with functional assessment of telomere length. Flow-FISH is considered the clinical standard for this purpose and provides confirmation of telomere shortening across specific leukocyte subsets, thereby supporting the molecular diagnosis and refining phenotype interpretation.

### 5.2. The Importance of Genetic Counselling in Clinical Practice

Based on the findings of this literature review, defining a structured diagnostic pathway under the guidance of a multidisciplinary team is essential to determine the most appropriate genetic test for each patient. Establishing a well-founded diagnostic suspicion not only optimizes the selection of molecular analyses but also reduces unnecessary expenditure of time and resources in identifying the underlying etiology. Once a molecular diagnosis is established, genetic counseling plays a pivotal role in providing patients and their families with clear information regarding the disease mechanism, prognosis, and recurrence risk. Counseling allows for informed decision-making and supports families in understanding the implications of the genetic findings.

For autosomal recessive disorders, parents should be informed that they may be healthy carriers and that each subsequent pregnancy carries a 25% recurrence risk, independent of fetal sex. Counseling should also address the availability of prenatal diagnosis and the possibility of medically assisted procreation (MAP) to manage reproductive risk. For autosomal dominant conditions, it is important to determine whether the identified variant is inherited or de novo. Segregation analysis in the parents clarifies carrier status and helps evaluate the potential contribution of incomplete penetrance. These considerations are particularly important when interpreting variants of uncertain significance (VUS), as assessing familial segregation can provide critical evidence for reclassification. Furthermore, integrating genetic findings with clinical, radiological, and functional data is often essential to refine their interpretation and avoid overestimating their diagnostic significance.

Overall, integrating genetic counselling into clinical practice ensures comprehensive family guidance, supports accurate risk assessment, and facilitates informed choices regarding management, surveillance, and reproductive planning.

## 6. Therapeutic Implications

### 6.1. Precision Medicine and Targeted Therapies

The management of ILDs has shifted from empirical, phenotype-based approaches to mechanism-driven strategies grounded in molecular genetics and disease-specific pathobiology. The chILD-EU consortium and subsequent European and North American statements have established a framework that integrates genetic, radiologic, and clinical data to guide diagnostic and therapeutic decisions, emphasizing multidisciplinary assessment referral to specialized centers, and biologically informed treatment strategies [2,7,87,92]. Within this framework, supportive measures such as oxygen supplementation, nutritional optimization, infection control, and prevention of pulmonary hypertension, remain the therapeutic cornerstone. Pharmacologic interventions are increasingly tailored to the dominant pathogenic mechanism rather than applied uniformly across diagnostic categories [12].

For inflammatory or organizing forms of chILD, ERS and ATS recommendations support systemic corticosteroids as first-line agents, followed when necessary by steroid-sparing immunosuppressants such as mycophenolate mofetil or azathioprine [12,87]. In immune-mediated and connective-tissue–disease–associated ILD, biologic and targeted immunomodulatory therapies are increasingly employed. Rituximab, abatacept, tocilizumab, belimumab, and other pathway-specific agents have shown encouraging results in pediatric cohorts, improving pulmonary function and facilitating glucocorticoid tapering, particularly in juvenile dermatomyositis–associated ILD and overlap syndromes [93]. Janus kinase (JAK) inhibitors such as ruxolitinib and baricitinib have become key therapeutic options for interferon-mediated and immune-driven ILDs, including COPA syndrome, SAVI, and TREX1- or IFIH1-related interferonopathies, by attenuating aberrant JAK–STAT signaling and type I interferon responses, with improvements in systemic inflammation, digital vasculopathy, and lung imaging [2,93]. These therapies require close hematologic and infectious monitoring, and interferon-stimulated gene expression profiling is increasingly used as a biomarker of therapeutic response.

Surfactant metabolism disorders exemplify the shift toward genotype-based management. Variants in *SFTPB*, *SFTPC*, and *ABCA3* represent the most frequent monogenic causes of neonatal or early-onset ILD, with clinical severity ranging from lethal neonatal respiratory failure to chronic fibrosing disease [12,21]. In *SFTPB* deficiency, where surfactant absence is irreversible, lung transplantation is the only curative option and should be considered early. In contrast, partially functional *SFTPC* and *ABCA3* variants may allow a period of medical stabilization, although treatment responses are highly variable and largely variant-specific. Recent series indicate that corticosteroids and hydroxychloroquine (HCQ) often provide only modest or transient benefit, particularly in misfolding-dominant *SFTPC* disease, where epithelial injury and endoplasmic-reticulum stress rather than reversible inflammation drive progression [17,19]. Case-based evidence also highlights mutation-specific HCQ efficacy: in an infant with a novel *SFTPC* variant (c.325–47_374del), early HCQ treatment failed to prevent fatal respiratory failure, underscoring that misfolding mechanisms may not be responsive to HCQ. Current expert opinion therefore recommends restricting HCQ to short, closely monitored trials and discontinuing it in the absence of early clinical improvement [94].

Telomere biology disorders, like other genetically determined forms of chILD, underscore the value of a mechanism-based therapeutic approach. In telomere-related disease, androgen therapy (e.g., danazol) may stabilize telomere length but carries hepatotoxic and thrombotic risks, necessitating strict monitoring and individualized risk–benefit assessment [95]. Emerging data also indicate that short telomere length in transplant candidates is associated with premature T-cell immunosenescence and increased early rejection risk, with implications for transplant timing and immunosuppressive protocols [95]. In interferonopathies, molecular confirmation of pathway activation is essential before initiating JAK inhibitors, given their toxicity profile and cost [2]. Overall, these developments converge on a unifying principle: in both genetic and immune-mediated pediatric ILD, early molecular and immunologic profiling is crucial to discriminate structural, misfolding-dominant, and immune-driven phenotypes, prioritize supportive care, avoid prolonged ineffective corticosteroid or cytotoxic exposure, and identify candidates for targeted biologic, antifibrotic, or transplantation strategies [12,96].

### 6.2. Impact of Genetic Diagnosis on Management Decisions

Genetic testing has fundamentally reshaped the management of pediatric ILDs by improving diagnostic precision, refining prognostic assessment, and informing therapeutic choices. Identification of a causal variant allows clinicians to distinguish inflammatory phenotypes, which may respond to immunomodulatory therapy, from structural or misfolding-related defects in anti-inflammatory agents are typically ineffective. In surfactant dysfunction disorders, genotype strongly influences both disease trajectory and treatment strategy: *SFTPB* loss-of-function variants mandate early referral for lung transplantation, whereas many *SFTPC* and *ABCA3* variants exhibit more heterogeneous courses and may justify time-limited trials of medical therapy before irreversible fibrotic remodeling occurs [12]. A recent case report of a compound heterozygous *ABCA3* mutation in a neonate with severe respiratory distress underscored how early molecular diagnosis can confirm a structural etiology, prevent futile escalation of corticosteroids or immunosuppressants, and support timely transplant planning [97].

Genetic information is equally critical in telomere biology disorders, in which pathogenic variants influence therapeutic decisions, transplant conditioning regimens, and long-term monitoring. Short telomere length and bone-marrow dysfunction increase the risk of hematologic toxicity, infection, and post-transplant complications, necessitating adapted conditioning protocols and careful immunosuppressive strategies [95]. Accordingly, identification of telomere-related gene variants in children has implications that extend beyond immediate therapeutic choices, informing longitudinal surveillance, family counseling, and risk stratification across generations. In this context, a pathway-based approach, rather than a focus on individual genes, allows integration of the overlapping pulmonary and extrapulmonary phenotypes associated with different telomere-related genes, supporting more coherent prognostic assessment and management planning [47]. Similarly, in interferonopathies such as COPA syndrome and STING1-associated disease, molecular confirmation supports the use of JAK inhibitors (ruxolitinib, baricitinib) to suppress the interferon signature and stabilize pulmonary involvement—an approach that would not be appropriate without documented pathway activation [2].

The first randomized, placebo-controlled phase 2 trial of HCQ in genetically confirmed chILD further underscores the importance of genotype-guided decision-making. In this chILD-EU multicenter crossover study, which included children carrying variants in *SFTPC*, *ABCA3*, *NKX2-1*, *TBX4*, and *COPA*, HCQ was well tolerated but did not significantly improve oxygen requirement, clinical respiratory scores, or growth compared with placebo, suggesting a limited and mechanism-specific benefit [98]. These findings support expert recommendations that HCQ and corticosteroids should be reserved for phenotypes with clear inflammatory components and used only in time-limited trials with predefined response criteria. They also reinforce the need for early genetic testing to avoid prolonged empirical therapy [99]. Neonatal management guidelines and national consensus statements echo this paradigm: in neonates with diffuse interstitial lung disease of suspected genetic origin, supportive care and exclusion of infectious or cardiac causes are prioritized, whereas prolonged corticosteroid or HCQ use is discouraged in the absence of demonstrable inflammation. Early molecular diagnosis and timely referral for transplantation are strongly encouraged [10].

Beyond therapy selection, genetic results guide longitudinal management, including timing of transplant referral, eligibility for targeted therapies, and surveillance strategies. Progressive fibrosing patterns, persistent oxygen dependence, or a forced vital capacity decline greater than 10% per year despite optimized care should prompt early multidisciplinary evaluation in transplant centers, particularly when high-risk genotypes or telomere-related defects are present [12]. Psychosocial support, family counseling, and structured transition programs for adolescents with chronic ILD integral components of this genotype-guided model of care [80,100]. Overall, the integration of genetic data into routine practice has transformed pediatric ILD from a largely empirical field to a precision-medicine discipline in which genotype-driven decision-making, multidisciplinary coordination, and proactive monitoring define the current standard of care [100].

### 6.3. Emerging Therapies and Clinical Trials

The therapeutic landscape of pediatric ILD is rapidly evolving, with antifibrotic agents, targeted immunomodulators, and experimental molecular therapies collectively redefining management paradigms. The most substantive advance to date is the introduction of antifibrotic therapy. The InPedILD trial demonstrated that nintedanib, a multitarget tyrosine-kinase inhibitor acting on VEGF, PDGF, and FGFR pathways, significantly reduced the rate of lung-function decline in children ≥ 6 years with progressive fibrosing ILD, with a safety profile comparable to that observed in adults [92]. Subsequent analyses and expert reviews have positioned nintedanib as the first disease-modifying drug for progressive pediatric ILDs regardless of genetic background, while highlighting the need for long-term surveillance of growth and hepatic function and the potential role of pirfenidone in mixed inflammatory–fibrotic phenotypes or in combination regimens [2,74].

Parallel progress in immune-mediated ILD has been driven by biologics and small-molecule inhibitors. Biologic agents targeting B- and T-cell pathways—rituximab, abatacept, tocilizumab, belimumab—have shown clinically meaningful improvements in pediatric connective-tissue–disease–associated ILD and overlap syndromes, enabling steroid tapering and stabilization of lung function [93]. For interferonopathies and autoinflammatory ILDs, JAK inhibitors provide a pathway-specific approach; pediatric series report improved systemic inflammation, better oxygenation, and radiologic stability with ruxolitinib or baricitinib, often combined with gradual glucocorticoid reduction. Dynamic monitoring of interferon-stimulated gene signatures is emerging as a tool for dose adjustment and early identification of responders [2,101]. These agents exemplify the shift from empirical immunosuppression toward mechanism-based immunomodulation in pediatric ILD.

Beyond pharmacologic modulation of inflammation and fibrosis, molecular and gene-based approaches aim to correct or compensate for underlying genetic defects. In surfactant dysfunction disorders, preclinical and early translational studies have identified chemical chaperones (e.g., 4-phenylbutyrate, carbamazepine) and autophagy enhancers (e.g., trehalose, rapamycin) as potential approaches to mitigate misfolding-induced cellular stress and restore proteostasis [92,102]. Patient-derived induced pluripotent stem cells (iPSCs) and lung organoids now allow functional testing of these compounds and evaluation of vector-based correction strategies in mutation-specific models [12]. Over the longer term, gene-editing and RNA-based therapies—including CRISPR/Cas9, base and prime editing, antisense oligonucleotides, and mRNA replacement—offer attractive possibilities for monogenic ILDs caused by *SFTPC*, *ABCA3*, or *SFTPB* variants. However, challenges related to delivery, safety, and long-term genomic stability currently confine these approaches to experimental settings [12,100].

International research networks and registries are critical to translating these advances into clinical benefit. The EU-funded “Orphans Unite: chILD Better Together” initiative created the first international registry and biobank dedicated to pediatric ILD, standardizing diagnostic protocols, centralizing data collection, and enabling multicenter trials [103]. The chILD-EU platform, together with ERS Clinical Research Collaborations, now underpins multi-omics integration, biobank-linked translational studies, and early-phase trials testing targeted therapies [7,92]. In summary, antifibrotic, immunomodulatory, and emerging gene-based therapies are progressively shifting pediatric ILD management away from predominantly supportive management toward disease-modifying, mechanism-based interventions defining a translational roadmap in which pediatric ILD serves as a model for precision therapeutics in rare respiratory disorders.

## 7. Impact of Genetic Findings on Prognosis

Genetic discoveries have fundamentally reshaped the diagnostic and prognostic landscape of chILD. Whereas these disorders were historically defined by clinical and radiologic features alone, molecular characterization now enables increasingly accurate predictions regarding disease severity, trajectory, and long-term outcomes. Understanding the genetic basis of chILD informs expectations for progression, therapeutic responsiveness, and extra-pulmonary involvement, and it supports anticipatory guidance for families, targeted monitoring strategies, and reproductive counseling. Genetic testing has therefore become a key tool for anticipating disease course. Beyond identifying the underlying pathogenic mechanism, the genotype often determines expected severity, clinical trajectory, and treatment responsiveness. The following examples illustrate how genetic characterization informs prognostic assessment across distinct forms of chILD.

Robust genotype–phenotype correlations are most clearly established among surfactant dysfunction disorders. Although many *SFTPC* variants are associated with relatively mild disease and favorable long-term survival, outcomes vary considerably with age at onset and variant type. In a retrospective analysis of 22 genetically confirmed cases (11 *SFTPC*, 5 *ABCA3*, 6 *NKX2-1*), important prognostic differences emerged [104]. Children with *NKX2-1* mutations had the earliest onset (median 0.4 years), the highest baseline respiratory burden, and the greatest prevalence of pulmonary hypertension (66.7%). In contrast, SFTPC-associated ILD presented with milder symptoms and no pulmonary hypertension. Serum KL-6 levels paralleled these patterns. Longitudinal outcomes diverged further: patients with *NKX2-1* variants experienced progressive respiratory decline, whereas those with *SFTPC* variants showed clinical, radiologic, and biomarker improvement. Mortality also reflected this gradient, with no deaths in the SFTPC cohort and 60% mortality in the *NKX2-1* group despite corticosteroid–hydroxychloroquine therapy.

Additional cohort analyses reinforce the heterogeneity of SFTPC-related disease. In an Argentine series of 14 children, neonatal respiratory distress was more frequent and more severe in ABCA3-related disease, while *SFTPC* variants generally conferred more favorable long-term outcomes [16]. Similarly, a Japanese cohort of 20 patients showed that neonatal-onset pulmonary alveolar proteinosis and specific high-risk variants (e.g., p.Leu45Arg or exon 4 splicing mutations) predict poor prognosis [20]. Collectively, these studies highlight the broad spectrum of SFTPC-associated ILD and the importance of variant-level risk stratification in prognostic assessment.

Although extremely rare, surfactant protein-B (SP-B) deficiency represents the most severe end of the surfactant dysfunction continuum. In a series of 11 genetically confirmed cases, complete loss-of-function variants caused fatal neonatal respiratory failure, whereas hypomorphic alleles permitting minimal residual SP-B expression allowed survival beyond infancy [105]. This contrast underscores how even small differences in protein function profoundly alter clinical trajectory. Similarly, the *ABCA3* genotype is a major determinant of prognosis, with clinical severity largely dependent on the degree of residual protein function. In a landmark cohort of 185 children, null/null genotypes caused severe neonatal respiratory failure with near-universal early mortality, whereas genotypes retaining partial function (null/other or other/other) were associated with variable presentation and significantly improved early survival [21]. Long-term data from a register-based cohort of 44 children surviving beyond the first year of life demonstrated that missense and small indel variants typically lead to chronic progressive ILD, though many patients survive into later childhood; absence of oxygen requirement was associated with longer survival [24]. These observations highlight the combined prognostic influence of variant type and early disease severity.

Variants in *FLNA* are increasingly recognized as causes of severe early-onset ILD. In a cohort of nine children, all required early respiratory support, three died in infancy from refractory respiratory failure and pulmonary hypertension, and survivors demonstrated persistent obstructive physiology with recurrent or ongoing pulmonary hypertension during follow-up [106]. Although some patients stabilize, FLNA-related ILD carries substantial early mortality and chronic cardiopulmonary morbidity. Finally, monogenic immune dysregulation disorders may present with progressive ILD, especially in late childhood or adolescence. These patients often have complex multisystem inflammatory phenotypes but lack a unifying diagnosis until genetic testing is pursued. Molecular confirmation is essential, as several conditions—such as SAVI—are amenable to precision therapies. In SAVI, initiation of JAK inhibitor therapy can halt or reverse ILD progression, demonstrating how early genetic identification directly improves prognosis [107].

## 8. Future Directions

### 8.1. Knowledge Gaps and Ongoing Research

Despite substantial progress in the classification and molecular characterization of pediatric ILDs, important gaps persist across diagnosis, pathobiology, and therapeutic evidence. The current ERS and ATS frameworks integrate clinical, radiologic, genetic, and pathological features, yet many children present with overlapping or atypical phenotypes that do not fit neatly into existing categories, and these schemes have not been rigorously validated in large, prospective pediatric cohorts [99]. Genotype–phenotype correlations for key surfactant- and transcription factor–related genes (e.g., *SFTPC*, *ABCA3*, *NKX2-1*, *TBX4*) remain incomplete, with considerable inter-individual variability in age of onset, radiologic pattern, and response to therapy, complicating prognostication and individualized treatment planning [102]. Radiologic assessment is limited by the absence of standardized HRCT scoring systems tailored to developmental anatomy and pediatric ILD patterns. Quantitative imaging and AI-based approaches show promise for objective assessment of disease burden and progression; these methods remain exploratory and unvalidated across centers [87].

Beyond genomic profiling, interest is increasingly directed toward circulating biomarkers of epithelial and alveolar injury, such as KL-6 and the surfactant proteins SP-A and SP-D, which may complement radiology and functional tests by reflecting epithelial stress and disease activity. As highlighted by Bush et al., however, their clinical utility in children remains exploratory due to limited pediatric data, non-standardized reference ranges and insufficient diagnostic specificity [74,87]. Therapeutic evidence remains a major unmet need. Most treatment strategies in pediatric ILD are extrapolated from adult data or rely on case series and retrospective cohorts, with limited randomized or prospective trials available to guide optimal drug selection, dosing, treatment duration, and monitoring protocols [12]. International registries have begun to clarify natural history and outcomes, but large-scale, disease-specific intervention studies are still lacking. Addressing these gaps will require sustained multicenter collaboration, standardized data collection, and closer integration of mechanistic research with clinical trial design.

### 8.2. Potential of Multi-Omics and Gene Editing

Multi-omics approaches offer an unprecedented opportunity to overcome many of the current limitations in pediatric ILD. While genomic sequencing has already transformed diagnosis, particularly for surfactant dysfunction and other monogenic disorders, the addition of transcriptomic, proteomic, metabolomic, and epigenomic data is beginning to delineate disease-specific stress responses, immune signatures, and fibrotic pathways that cannot be fully appreciated through imaging or histopathology alone [108]. A further strength of multi-omics lies in the possibility of integrating heterogeneous datasets through advanced computational tools. Machine-learning models can merge genomic, transcriptomic, proteomic and metabolomic layers to identify molecular endotypes, infer dynamic disease trajectories and prioritize therapeutic targets that remain invisible to phenotype-based classification. These integrative strategies depend on harmonized biobanks and international registries, a key priority emphasized in recent consensus efforts, and could ultimately support individualized risk stratification when combined with quantitative HRCT metrics and longitudinal clinical data [100]. Yet substantial challenges persist, including small sample sizes, developmental variability, limited access to serial biospecimens and the absence of validated omics-based classifiers for routine decision-making.

In parallel, patient-derived iPSCs and lung organoids provide complementary functional platforms in which patient-specific mutations can be modeled and candidate therapies—such as autophagy modulators, chaperone-based treatments, and antifibrotic agents—can be tested under controlled conditions [109]. These systems enable rapid assessment of drug toxicity and efficacy, facilitate the study of rare genotypes, and offer a bridge between bench discoveries and early-phase clinical trials.

Gene editing and RNA-based therapies represent the most ambitious frontier. Technologies such as CRISPR/Cas9, base editing, and prime editing offer proof-of-concept for correcting pathogenic variants in surfactant and telomere-related genes, while antisense oligonucleotides and mRNA replacement therapies may allow transient or partial functional rescue [92]. However, major challenges remain, including efficient and safe delivery to the distal airways, avoidance of off-target effects, long-term genomic stability, and ethical considerations surrounding germline and pediatric interventions. For the foreseeable future, these approaches are likely to remain confined to experimental models and highly selected early-phase trials, but they nonetheless provide a conceptual framework for curative strategies in monogenic pediatric ILDs [12]. Consistent with this translational perspective, in monogenic chILD forms related to *CSF2RA* or *CSF2RB* variants, which impair GM-CSF receptor function and lead to an alveolar proteinosis–like phenotype, emerging therapeutic strategies aim to restore GM-CSF signaling and alveolar macrophage function, building on insights from reported familial cases [29,30].

### 8.3. Personalized Medicine in Pediatric ILDs

Personalized medicine is emerging as the future paradigm of pediatric ILD care, integrating genetic, molecular, radiologic, physiologic, and digital-health data into individualized management plans. Genotype-guided strategies in surfactant dysfunction disorders illustrate this need: variability in clinical presentation and trajectories among *SFTPC*, *ABCA3*, and *NKX2-1* variants—well documented across international cohorts—highlights the importance of precise molecular diagnosis and early stratification [104]. Misfolding-prone *SFTPC* variants may benefit from chaperone or autophagy-targeted therapies, whereas *ABCA3* loss-of-function variants often progress to fibrosis, warranting earlier consideration of immunomodulatory or antifibrotic agents and timely evaluation for transplantation [102]. Beyond canonical surfactant-related genes, emerging variants of uncertain significance—such as *FLNA* and *ARHGAP42*—underscore the need for mechanistic studies capable of defining actionable pathways. While no gene-specific therapies are currently available for these disorders, potential future avenues may include pathway-targeted interventions, personalized immunomodulatory approaches, and, in the long term, gene- or transcript-editing strategies; however, these possibilities remain preliminary and require functional validation before clinical translation.

Similarly, for genes implicated in embryonic lung development and transcriptional regulation (such as *TBX4*, *FOXF1*, and *FGF10*), no targeted or gene-specific therapies are currently available and current management therefore remains supportive and focused on complications [70,75]. Nevertheless, advancing mechanistic insights into disrupted developmental and signaling pathways may eventually provide a conceptual foundation for future mechanism-based or gene-editing strategies.

Achieving truly personalized medicine will depend on the convergence of three elements: (1) routine genome-wide diagnostics with rapid turnaround for suspected chILD; (2) interoperable international registries integrating longitudinal clinical and multi-omic datasets; and (3) translational platforms such as patient-derived organoids, iPSC-derived AT2 cells, and in vivo models that enable functional validation and preclinical drug screening [100]. These components collectively support biomarker discovery, adaptive trial designs, and the transition of monogenic chILD from supportive management to targeted or disease-modifying therapies. Integrated multimodal data streams—including biomarkers, quantitative HRCT metrics, home spirometry, wearable sensors, and remote auscultation—may further enable early detection of exacerbations and more precise therapy titration, while psychosocial factors remain essential for shared decision-making [74]. Evaluation of emerging techniques, such as multiple-breath washout, may further refine disease monitoring and aid clinical management in selected patients [110,111,112]. International collaboration is essential: the European chILD registry and similar initiatives provide platforms for biomarker validation, refinement of classification systems, and the development of genotype- and pathway-specific trials [7,100,103]. The integration of multi-omics, advanced imaging, digital health technologies, and multinational registries is expected to embed personalized medicine into routine care and advance pediatric ILD toward a truly mechanism-driven discipline.

## 9. Conclusions

Genetic testing plays a central role in the management of chILD, linking clinical expression to the underlying biology. Molecular diagnosis enables accurate prognostic stratification, supports individualized follow-up, and informs targeted therapeutic strategies within a precision-medicine framework. A confirmed genetic diagnosis is also essential for providing families with accurate recurrence-risk counseling and for planning timely care in future pregnancies. Furthermore, integrating genomic testing with multi-omics approaches, functional models, and international registries paves the way toward predictive and personalized medicine. Early integration of comprehensive molecular diagnostics into clinical evaluation is therefore crucial to refine disease classification, bridge current knowledge gaps, and advance the management of pediatric ILD toward an etiological mechanism-driven framework.

## Figures and Tables

**Table 1:** Selected genes associated with isolated and syndromic forms of chILD.

	Functional Impact	Gene	Inheritance	Onset	Related Phenotype	Key ILD Features
**Isolated chILDs**	**Surfactant-related disorders**	*SFTPC*	AD (usually de novo)	Neonatal–childhood	Surfactant metabolism dysfunction, pulmonary, 2	Ground-glass pattern, Alveolar proteinosis
		*SFTPB*	AR	Neonatal	Surfactant metabolism dysfunction, pulmonary, 1	Ground-glass pattern, Alveolar proteinosis, Minimal exogenous surfactant response
		*ABCA3*	AR	Neonatal–childhood (rare later-onset)	Surfactant metabolism dysfunction, pulmonary, 3	Ground-glass pattern, Progressive fibrosis Lamellar body deposition,
		*SFTPA1/SFTPA2*	AD	Adult/rare pediatric	Interstitial lung disease 1, 2	Pulmonary fibrosis, Early lung cancer risk
	**GM-CSF receptor-related disorders**	*CSF2RA*	PAR	Childhood	Surfactant metabolism dysfunction, pulmonary, 4	Ground-glass pattern, Defective alveolar macrophage maturation
		*CSF2RB*	AR	Adult/rare pediatric	Surfactant metabolism dysfunction, pulmonary, 5	Ground-glass pattern, Defective alveolar macrophage maturation
	**Lysosomal-related disorders**	*LAMP3*	AR	Childhood	–	Ground-glass pattern, Pulmonary fibrosis, Alveolar cell hyperplasia
**Syndromic chILDs**	**Telomere maintenance**	*TERT/TERC*	AD	Childhood-adult	Pulmonary fibrosis and/or bone marrow failure syndrome, telomere-related 1, 2	Pulmonary fibrosis
		*NOP10/TINF2*	AR/AD	Adolescence/ childhood-adult	Dyskeratosis congenita, autosomal recessive 1, autosomal dominant 3	Pulmonary fibrosis
		*DKC1*	X-linked	Childhood-adolescence	Dyskeratosis congenita, X-linked	Pulmonary fibrosis
	**Immune disorders/interferonopathies**	*STING1*	AD	Childhood	STING-associated vasculopathy with onset in infancy	Ground-glass pattern, Pulmonary fibrosis, Alveolar macrophage infiltration
		*COPA*	AD	Adolescence-young adult	Autoinflammation and autoimmunity, systemic, with immune dysregulation 1	Ground-glass pattern, Lung cysts, Alveolar hemorrhage
		*STAT5B*	AR	Childhood	Growth hormone insensitivity with immune dysregulation 1	Pulmonary fibrosis
		*OAS1*	AD	Neonatal	Immunodeficiency 100 with pulmonary alveolar proteinosis and hypogammaglobulinemia	Lung consolidations, Alveolar proteinosis
		*CCR2*	AR	Childhood	Polycystic lung disease	Lung cysts, Alveolar proteinosis, Mild interstitial fibrosis
	**Cytoskeletal/structural disorganization**	*ITGA3*	AR	Neonatal	Epidermolysis bullosa junctional 7 with interstitial lung disease and nephrotic syndrome	Interstitial lung disease
	**Pulmonary development/transcriptional dysregulation**	*NKX2-1*	AD	Neonatal	Choreoathetosis, hypothyroidism, and neonatal respiratory distress	Interstitial lung disease, Pulmonary fibrosis
		*TBX4*	AD	Childhood	Ischiocoxopodopatellar syndrome, with or without pulmonary arterial hypertension	Acinar dysplasia
	**Aminoacyl-tRNA synthetase disorders**	*FARSA/FARSB*	AR	Childhood	Rajab interstitial lung disease with brain calcifications 1, 2	Pulmonary fibrosis, Alveolar proteinosis
		*MARS1*	AR	Childhood	Interstitial lung and liver disease	Pulmonary fibrosis, Alveolar proteinosis
		*YARS1*	AR	Childhood	Infantile-onset multisystem neurologic, endocrine, and pancreatic disease 2	Lung cysts, Pulmonary fibrosis
	**Multisystem fibrosing syndromes**	*NHLRC2*	AR	Childhood	FINCA syndrome	Pulmonary fibrosis
		*FAM111B*	AD	Childhood	Poikiloderma, hereditary fibrosing, with tendon contractures, myopathy, and pulmonary fibrosis	Pulmonary fibrosis

This table includes genes for which a well-established association with pediatric interstitial lung disease has been reported. Genes discussed in the manuscript text with emerging, preliminary, or indirect evidence were intentionally not included.

## Data Availability

No new data were created or analyzed in this study. Data sharing is not applicable to this article.

## Supplementary Materials

The following supporting information can be downloaded at: https://www.mdpi.com/article/10.3390/biomedicines14020385/s1, Search strategies and results.

## Abbreviations

The following abbreviations are used in this manuscript:

chILDs	Children’s interstitial lung diseases
CTD-ILD	Connective tissue disease–associated ILD
NEHI	Neuroendocrine cell hyperplasia of infancy
DIP	Desquamative interstitial pneumonia
NSIP	Non-specific interstitial pneumonia
HRCT	High-Resolution Computed Tomography
IPF	Idiopathic pulmonary fibrosis
VATS	Video-assisted thoracoscopic surgery
MDD	Multidisciplinary diagnostic discussions
SPC	Pulmonary-associated surfactant protein C
SPB	Surfactant protein B
snoRNP	small nucleolar ribonucleoprotein
SAVI	STING-associated vasculopathy with onset in infancy
COPI	Coatomer protein complex I
GH	Growth hormone
GRAF	GTPase Regulator Associated with Focal Adhesion Kinase
FINCA	FIbrosis, Neurodegeneration and Cerebral Angiomatosis
CNVs	Copy Number Variations
PIG	Pulmonary interstitial glycogenosis
CT	Computed Tomography
VATS	Video-assisted thoracoscopic
UIP	Usual interstitial pneumonia
CAD	Congenital alveolar dysplasia
AD	Acinar dysplasia
ACD	Alveolocapillary dysplasia
MPV	Misalignment of pulmonary veins
CPI	Chronic pneumonitis of infancy
LIP	Lymphocytic interstitial pneumonia
PAP	Pulmonary alveolar proteinosis
MRI	Magnetic resonance imaging
PAS	Periodic acid–Schiff
BAL	Bronchoalveolar lavage
NGS	Next-generation sequencing
WES	Whole-exome sequencing
WGS	Whole-genome sequencing
CMA	Chromosomal microarray
FISH	Fluorescence in situ hybridization
CADDS	Contiguous ABCD1/DXS1357E deletion syndrome
VUS	Variants of uncertain significance
ERS	European Respiratory Society
ATS	American Thoracic Society
LoF	Loss of Function
AR	Autosomal Recessive
AD	Autosomal Dominant
PAR	Pseudoautosomal Recessive
GoF	Gain of Function
