# Supplementary material for: The Impact of Genetics on Pediatric Interstitial Lung Diseases: A Narrative Literature Review and Clinical Implications
Source: Biomedicines. 2026 Feb 6;14(2):385. doi: 10.3390/biomedicines14020385 (PMC12938008; doi:10.3390/biomedicines14020385)
Supplement: Supplementary file 1 [file biomedicines-14-00385-s001.zip › biomedicines-4113283-supplementary.pdf]

Search strategy

We searched the published literature on 23 September 2025

**PubMed – n = 409**
("child*" OR "pediatric*" OR "infant*" OR "adolescent*") AND ("interstitial lung disease*" OR "ILD" OR "pulmonary fibrosis" OR "diffuse parenchymal lung disease*") AND ("genetic*" OR "mutation*" OR "genotype*" OR "molecular diagnosis" OR "next-generation sequencing" OR "NGS" OR "genetic testing")<br><br>Filters: Language: English; Age: Child: birth-18 years; Publication years: 2015-2025
***EMBASE* – n = 910**
('child'/exp OR child*:ti,ab,kw OR pediatric*:ti,ab,kw OR infant*:ti,ab,kw OR adolescent*:ti,ab,kw) AND ('interstitial lung disease'/exp OR 'pulmonary fibrosis'/exp OR 'diffuse parenchymal lung disease':ti,ab,kw OR 'interstitial lung disease*':ti,ab,kw OR ild:ti,ab,kw OR 'pulmonary fibrosis':ti,ab,kw) AND ('genetics'/exp OR 'mutation'/exp OR 'genotype'/exp OR 'genetic testing'/exp OR 'next generation sequencing'/exp OR genetic*:ti,ab,kw OR mutation*:ti,ab,kw OR genotype*:ti,ab,kw OR 'molecular diagnosis':ti,ab,kw OR 'next-generation sequencing':ti,ab,kw OR ngs:ti,ab,kw OR 'genetic testing':ti,ab,kw)<br><br>Filters:<br><br>Age: Newborn (0-1 month); Infant (1-12 months); Child (1-12 years); Preschool child (1-6 years); School child (7-12 years)4; Adolescent (13-17 years)<br><br>Publication years: 2015-2025<br><br>Language: English
